# Distinct Structure of Cortical Population Activity on Fast and Infraslow Timescales

**DOI:** 10.1093/cercor/bhz023

**Published:** 2019-02-23

**Authors:** Michael Okun, Nicholas A Steinmetz, Armin Lak, Martynas Dervinis, Kenneth D Harris

**Affiliations:** 1 Centre for Systems Neuroscience and Department of Neuroscience, Psychology and Behaviour, University of Leicester, Leicester, UK; 2 Institute of Neurology, University College London, London, UK

**Keywords:** neural dynamics, phase coupling, power-law, resting state fMRI, scale-free dynamics

## Abstract

Cortical activity is organized across multiple spatial and temporal scales. Most research on the dynamics of neuronal spiking is concerned with timescales of 1 ms–1 s, and little is known about spiking dynamics on timescales of tens of seconds and minutes. Here, we used frequency domain analyses to study the structure of individual neurons’ spiking activity and its coupling to local population rate and to arousal level across 0.01–100 Hz frequency range. In mouse medial prefrontal cortex, the spiking dynamics of individual neurons could be quantitatively captured by a combination of interspike interval and firing rate power spectrum distributions. The relative strength of coherence with local population often differed across timescales: a neuron strongly coupled to population rate on fast timescales could be weakly coupled on slow timescales, and vice versa. On slow but not fast timescales, a substantial proportion of neurons showed firing anticorrelated with the population. Infraslow firing rate changes were largely determined by arousal rather than by local factors, which could explain the timescale dependence of individual neurons’ population coupling strength. These observations demonstrate how neurons simultaneously partake in fast local dynamics, and slow brain-wide dynamics, extending our understanding of infraslow cortical activity beyond the mesoscale resolution of fMRI.

A single action potential lasts about a millisecond, and a second suffices for a vast range of sensory-motor and cognitive behaviors, such as recognizing pictures and sounds, getting up or sitting down, or recalling a memory. Accordingly, most neurophysiological research has focused on subsecond timescales. However, several neural processes occur over much longer timescales (Huk et al. 2018). Transitions between sleep and wakefulness and between different stages of sleep occur on timescales of minutes and hours (Weber and Dan 2016; Lecci et al. 2017; Meisel et al. 2017). During wakefulness, changes in arousal can span tens of seconds and minutes, yet they affect performance in subsecond behavioral tasks (Harris and Thiele 2011; Palva and Palva 2012; McGinley et al. 2015). Slow timescale dynamics has been revealed by resting-state fMRI (Raichle 2015), which infers neural activity from the (slow) changes in blood supply to different areas of the brain. However, fMRI monitoring of neural activity is limited to the so called infraslow range of 0.01–1 Hz. Furthermore, both fMRI and other approaches to study mesoscale infraslow cortical dynamics—such as electro- and magneto-encephalography (EEG, ECoG, LFP, MEG, e.g., see Popa et al. 2009; Palva and Palva 2012; Mitra et al. 2018), and intrinsic and voltage-sensitive fluorescent-protein or dye imaging in experimental animals (White et al. 2011; Chan et al. 2015; Kraft et al. 2017)—cannot characterise individual neurons’ relationships to infraslow activity.

The relationship of individual neurons to infraslow brain dynamics, and the relationship between a neuron’s coupling to infraslow and fast mesoscale cortical dynamics, is thus poorly understood. For example, how much can the firing rate of an individual neuron change over tens of seconds and minutes, and how can these slow dynamics be summarized quantitatively? To what extent are slow changes in firing rate correlated among neurons, and what is the structure of these slow correlations? To what extent are neurons’ relationships to slow and fast firing rate fluctuations similar, and might they be driven by the same underlying mechanisms?

Here we addressed these questions by analyzing multihour recordings of neuronal populations in mouse medial prefrontal cortex (mPFC), performed using chronically implanted high-density silicon probes. We found that neuronal spike trains have 1/f power spectral density (PSD), and that PSD in combination with interspike interval (ISI) distribution suffices for an accurate quantitative model of single neuron spiking dynamics on both fast and slow timescales. Coupling between individual neurons and the population rate (defined as the summed rate of all the spikes recorded by the probe) was timescale-dependent, with many neurons strongly coupled to population rate on fast timescales but weakly coupled on slow timescales, or vice versa. Furthermore, on slow but not fast timescales, neurons’ phase preference with respect to the population rate was bimodal. Finally, in frequencies ≤0.1 Hz population rate was highly correlated with arousal as reflected by the pupil area. These results suggest that dynamics on fast and infraslow timescales are distinct processes, and likely regulated by distinct mechanisms at the single neuron level.

## Materials and Methods

### Electrophysiological Recordings

All experimental procedures were conducted according to the UK Animals (Scientific Procedures) Act 1986 (Amendment Regulations 2012). Experiments were performed at University College London under personal and project licenses released by the Home Office following institutional ethics review. Adult C57BL/6 mice of both sexes were used.

The experimental procedures for chronically implanting Neuronexus and Neuropixels probes were previously described in (Okun et al. 2016; Jun et al. 2017). Briefly, in an initial surgery under isoflurane anesthesia animals were implanted with a custom built head-plate. Following full recovery and acclimatization to head-fixation, probe implantation was performed under isoflurane anesthesia. The probes were implanted through a craniectomy above mPFC (0.5 mm lateral and 1.8 mm anterior to bregma). Neuronexus probes (A2 × 2-tet with CM16LP connector and Buzsaki32 with CM32 connector) were lowered 1.7 mm, placing the recording sites in the prelimbic cortex (PrL). Neuropixels probes were lowered ~3.5 mm (so that the most superficial of the 374 recording sites remained outside of the brain, while the deepest sites were ~3.5 mm inside the brain; the recording sites were thus placed in the cingulate, prelimbic, and infralimbic cortices). The probes were oriented approximately parallel to the cortical layers, ~0.5 mm lateral offset of the insertion point relative to the midline implied that the probes resided in cortical layers 5 and 6 (which was also confirmed histologically).

Recordings were performed over the course of several months following the probe implantation. For head-fixed recording, mice were placed inside a plastic tube where they could comfortably sit or stand. The recordings lasted 1.5–3 h. In animals implanted with a Neuronexus probe, recordings were performed using OpenEphys (www.open-ephys.org) recording system (Siegle et al. 2017). Mice with a Neuropixels probe were recorded using SpikeGLX system (github.com/billkarsh/SpikeGLX) developed at Janelia Farm. (Some of the mice were trained and recorded in a behavioral task which the animals would perform for water reward (Lak et al. 2018); the data analyzed here is from recordings of ongoing activity in separate sessions without behavior, performed on days when the animals were not water deprived).

Recordings in freely behaving animals implanted with a Neuronexus probe lasted 4–8 h. Mice were briefly head-fixed to allow attaching the amplifier head-stage to the probe and then released into their home cage, where they were free to engage in any activity of their choice, while being monitored to make sure that the thin cable leading from the amplifier to the OpenEphys box was not entangled.

### Spike Sorting and Drift Contamination

Spike sorting of Neuropixels recordings was performed using Kilosort software (Pachitariu et al. 2016), with manual curation performed using phy (github.com/cortex-lab/KiloSort and github.com/kwikteam/phy). Spike sorting of Neuronexus probe recordings was performed similarly, or using SpikeDetekt, KlustaKwik, and Klustaviewa software suite (Rossant et al. 2016).

We have evaluated the quality of spike sorted units using isolation distance metric (Schmitzer-Torbert et al. 2005) and by quantifying the contamination of the refractory periods of the spike autocorrelograms, which was expressed as proportion of the number of spikes in the first 2 ms of the autocorrelogram relative to the autocorrelogram asymptote (Harris et al. 2000). We have limited the analysis to units with isolation distance > 20 and refractory period contamination < 0.2. Our analyses yielded quantitatively similar results when more (and less) stringent criteria were applied.

A possible concern is that our results, instead of reflecting the properties of actual infraslow fluctuations in the firing rates of cortical neuronal populations, are dominated by contamination introduced by unstable recordings. Such concern is not unique to the present work, and was raised in the past regarding estimation of pairwise correlations (Ecker et al. 2010). Here, Neuropixels recordings provided an unprecedented opportunity to detect and monitor drifts, as the recording sites span a contiguous stretch of >3 mm. For spikes detected simultaneously on several contacts, we have computed the vertical location of the “center of mass” of the spike, according to the relative amplitude of the spike waveform on each contact. Changes in these locations over time, particularly for high-amplitude spikes, reveal potential drifts. In the example shown in Fig. S1, multiple drift events are visually apparent. In each event, the vertical locations of high-amplitude spikes at a particular neighborhood of the probe drift ~10 μm upwards over the course of ~5 s, and over the next ~20–40 s return to their original locations. Similar drift pattern occurs ~200 μm further down the shank (Fig. S1b,c), which is a strong indication that these drifts are produced by a vertical movement of the probe with respect to cortical tissue, rather than any other cause. In fact, drifts were simultaneously observed at >10 locations across the top 800 μm of the probe. Drifts were not observed when vertical location of low-amplitude spikes was considered. This is consistent with the idea that a vertical movement has a much larger impact on the waveforms of high-amplitude spikes originating from neurons abutting the probe, compared with the low-amplitude spike waveforms of neurons that are more (horizontally) distant.

In the example recording, drifts of high-amplitude spikes were not observed on contacts deeper than ~800 μm (Fig. S1d). In recordings from this mouse, all data originating from the top 1 mm was excluded from the analyses. No similar drifts were observed in Neuropixels recordings from the second animal. In recordings performed with Neuronexus probes, the recording sites were located only at the bottom 200 μm of probes which were lowered 1.7 mm into the brain, thus to the extent that drifts of Neuropixels and Neuronexus probes are similar, we do not expect to find vertical drifts in these recordings (since the recording sites were not covering a contiguous interval, the above drift analysis cannot be repeated for Neuronexus recordings).

An additional observation suggesting that our results are not driven by drifts concerns the relationship between amplitude of the different units and their phase preference. If drifts introduce a strong bias into our estimation of phase with respect to population rate (see below), then there might exist some consistent relationship between the phase and spike waveform amplitude of the different units, because drift bias is expected to be stronger for units close to the probe and having high-amplitude spike waveforms. However, no significant correlation between phase and spike amplitude was found in our data. We conclude that drift is an important caveat that has the potential to bias measurements of spiking activity obtained with extracellular probes, however in view of the control analyses explained above, we believe that the phenomena described here are not due to such drifts.

### Pupil Tracking

Pupil area was tracked as previously described in Burgess et al. (2017). Briefly, a camera (DMK 21BU04.H or DMK 23U618, The Imaging Source) with a zoom lens (ThorLabs MVL7000) was focused on one of the eyes of the animal. The eye was illuminated by an infrared LED (SLS-0208A, Mightex). Videos of the eye were acquired at ≥30 Hz. In each video frame, excluding frames with blinks, an ellipse was fit to the pupil image, and pupil area was estimated based on this fit.

### Single Spike Train Analysis

PSD of individual spike trains (Fig. 2*a*) was estimated using mtspectrumsegpb function of Chronux toolbox (chronux.org). Note that this function is specifically intended for point processes such as spike trains (Mitra and Bokil 2007). PSD in different frequencies was estimated by breaking the entire recording into segments of appropriate length, and averaging across them. Specifically, segments of length l were used to estimate PSD in 7/l to 10/l frequencies (for example, a 2 h recording is broken into 72 000 segments, 0.1 s each, and these segments are used to estimate PSD in 75–100 Hz frequencies. Then the recording is broken into 54 014 segments, 0.133 s each, to estimate PSD in 56–75 Hz frequencies, and so on. Eventually 12 segments of 556 s are used to estimate PSD in 0.013–0.018 Hz frequencies). For frequencies <0.01 Hz the spectrum of the entire recording was computed using mtspectrumpb function without breaking it into segments. For presentation purposes only it was further smoothed using Matlab’s smooth function.

**Figure 1.**
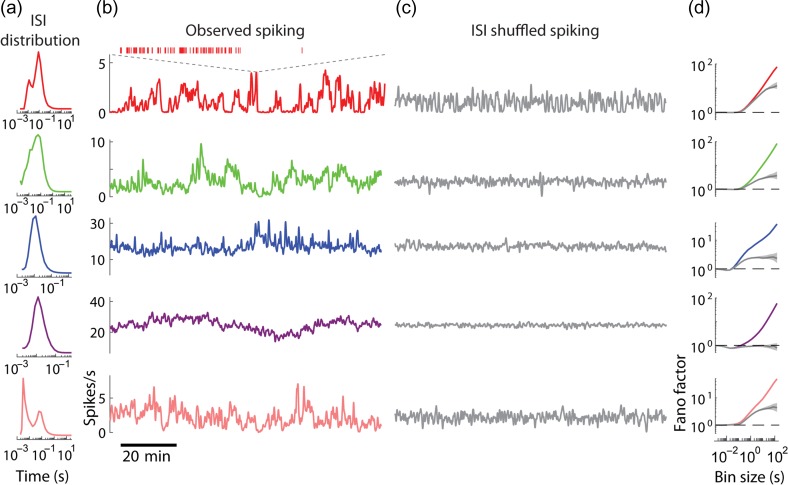
Fast and slow timescale dynamics of individual cortical neurons. (*a*) ISI distribution of 5 simultaneously recorded example neurons in mPFC of an awake, head-fixed mouse. (*b*) Firing rate (smoothed with 8 s FWHM Gaussian) over the course of the recording for the neurons in *a* (inset: spike train corresponding to the shaded region in the red trace). (*c*) Firing rate of ISI-shuffled spike trains (cf. *b*). (*d*) Fano factors of spike counts using bins of 10^−3^ –10^2^ s for original (color) and ISI-shuffled (gray) spike trains. Shaded areas indicate 95% confidence intervals.

**Figure 2.**
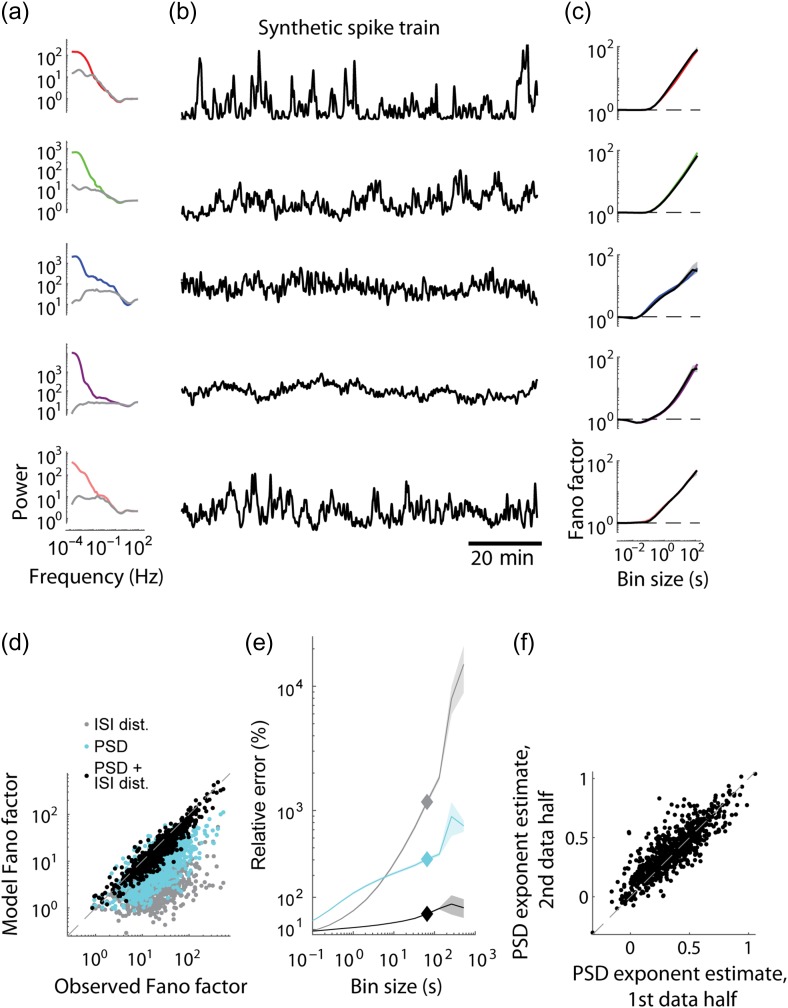
Modeling spiking dynamics on fast and slow timescales. (*a*) PSD of the original (color) and ISI-shuffled (gray) spike trains for the 5 example neurons shown in Figure 1. (*b*) Firing rate of synthetic spike trains constructed by requiring that their ISI distribution and PSD match the original data (cf. Fig. 1*b*,*c*). (*c*) Fano factors of spike counts using bins of 10^−3^–10^2^ s: the plots for original data (color) and for synthetic spike trains (black) closely match (cf. Fig. 1*d*). Shaded areas (where visible) indicate 95% confidence intervals. (*d*) Observed and predicted spike count Fano factors for 65 s bins for the entire dataset (775 neurons). Predictions were based on ISI distribution only (gray), on PSD only (cyan), or on the full model in which both constraints apply (black). (*e*) Relative error (in %) of predicting the observed Fano factors for bins of 10^−3^–10^2^ s for the 3 models, averaged over all neurons. Diamonds mark values for 65 s bin, shown in *d* (1072%, 305%, and 47% errors of ISI only, PSD only and the full models, averaged across all neurons). Shaded area shows the standard error. (*f*) The PSD of each neuron was fit with a $\mathrm{const}/f^{\beta }$ function in the range 0.01–1 Hz. The power-law exponent *β* is specific to each neuron, which is demonstrated by the fact that the values estimated separately in 2 halves of the recording closely match (*R*^2^ = 0.69, *P* < 10^−100^).

PSD of a spike train is closely related to, but distinct from, the PSD of the underlying (continuous) firing rate intensity. If the firing rate λ(t) is itself a random process with power spectrum $S_{\lambda \lambda }\left(\omega \right)$, then the power spectrum of the spike train $S_{\mathrm{nn}}\left(\omega \right)\approx \mu_{\lambda }+S_{\lambda \lambda }\left(\omega \right)$, where $\mu_{\lambda }$ is mean firing rate (Lepage et al. 2011). To intuitively see why the $\mu_{\lambda }$ term in the right hand side of the equation is required, consider the simplest case where λ(t)=const, that is, the case of a constant intensity (homogeneous) Poisson spike train stochastic process. In this case $S_{\lambda \lambda }\left(\omega \right)=0$, and $S_{\mathrm{nn}}\left(\omega \right)$ = $\mu_{\lambda }$. A homogeneous Poisson spike train has constant power in all frequencies for the same reason that white noise has power in all frequencies. The power spectrum is the Fourier transform of the autocorrelation (a result known as the Wiener–Khinchin theorem). The autocorrelation of a Poisson spike train is a delta function at time 0, because of the correlation of each spike with itself, and is zero at all other times since no other spikes are correlated. The Fourier transform of a delta function is a constant function, explaining why the power spectrum of a Poisson spike train is flat.

### Single Spike Train Modeling

The spike train model which captures both the fast and slow timescale dynamics of cortical spiking relies on both ISI distribution and PSD of spike trains (Fig. 2). The goal of the model is to generate synthetic spike trains satisfying constraints on both distributions simultaneously.

Let I denote the observed ISI distribution of a spike train. For modeling, I was represented by a histogram with logarithmically spaced bins of all the observed ISIs (32 bins were used to describe ISIs, from 1 ms up to 200 s). Instead of using the PSD of the spike train itself, the model uses the PSD of the underlying continuous firing rate intensity, which we denote by ℘ (the two are closely related but distinct, as described above). For modeling, ℘ was represented by the power of a continuous signal obtained by convolving the observed spike train with a 50 ms FWHM Gaussian (50 parameters were used to represent ℘). With I and ℘ as its inputs (82 parameters in total), the goal of the model is to generate a synthetic spike train whose ISI distribution and PSD are as close as possible to the original spike train. An intermediate step towards this final goal is constructing a continuous firing rate intensity signal r(t) for the synthetic spike train. However, we start by constructing a different firing rate intensity signal, $r_{1}\left(t\right)$, by sampling ISIs from I and convolving the resulting spike train with a 50 ms FWHM Gaussian. Typically $r_{1}\left(t\right)$ will have much less power in the infraslow frequencies than what is required. On the other hand, the straightforward way to generate a signal with power ℘ (i.e., to use inverse Fourier transform) produces a signal whose values are normally distributed with 0 mean, which is inappropriate for a firing rate intensity function. Therefore, we used an iterative algorithm of (Schreiber and Schmitz 1996) to generate a signal r(t) with power ℘ and distribution of values of $r_{1}\left(t\right)$. Once r(t) was generated, we sample a spike train $n_{1}$ using r(t) as a time dependent firing rate signal. In the final step the ISIs of the spike train are adjusted to have the desired distribution I. Specifically, we convert the sequence of ISIs in $n_{1}$ into a sequence of ISI ranks, by replacing each ISI with its rank among all the ISIs of $n_{1}$. We build the final output spike train n by sampling from I the same number of ISIs found in $n_{1}$ and rearranging them according to the sequence of ranks from $n_{1}$, that is, the ISI rank sequences of n and of $n_{1}$ match.

When the model is used to generate an output without an explicit constraint on I (i.e., only ℘ input is provided), it implicitly assumes I has an exponential distribution, with an additional constraint forbidding ISIs <2 ms (representing a hard refractory period).

Although it is possible in principle to consider a spike train model which relies solely on the autocorrelation structure (equivalently the PSD), these models are complex and not necessary for our purposes. The reason we used ISIs to model fast timescale dynamics and PSD for slow timescale dynamics is that ISI distribution provides a simple generative model for fast timescale dynamics of spike trains, whereas methods for constructing spike trains with a given (fast timescale) autocorrelation are substantially more complex (Krumin and Shoham 2009; Macke et al. 2009).

### Time Domain Population Coupling on Fast and Slow Timescales

**Figure 3.**
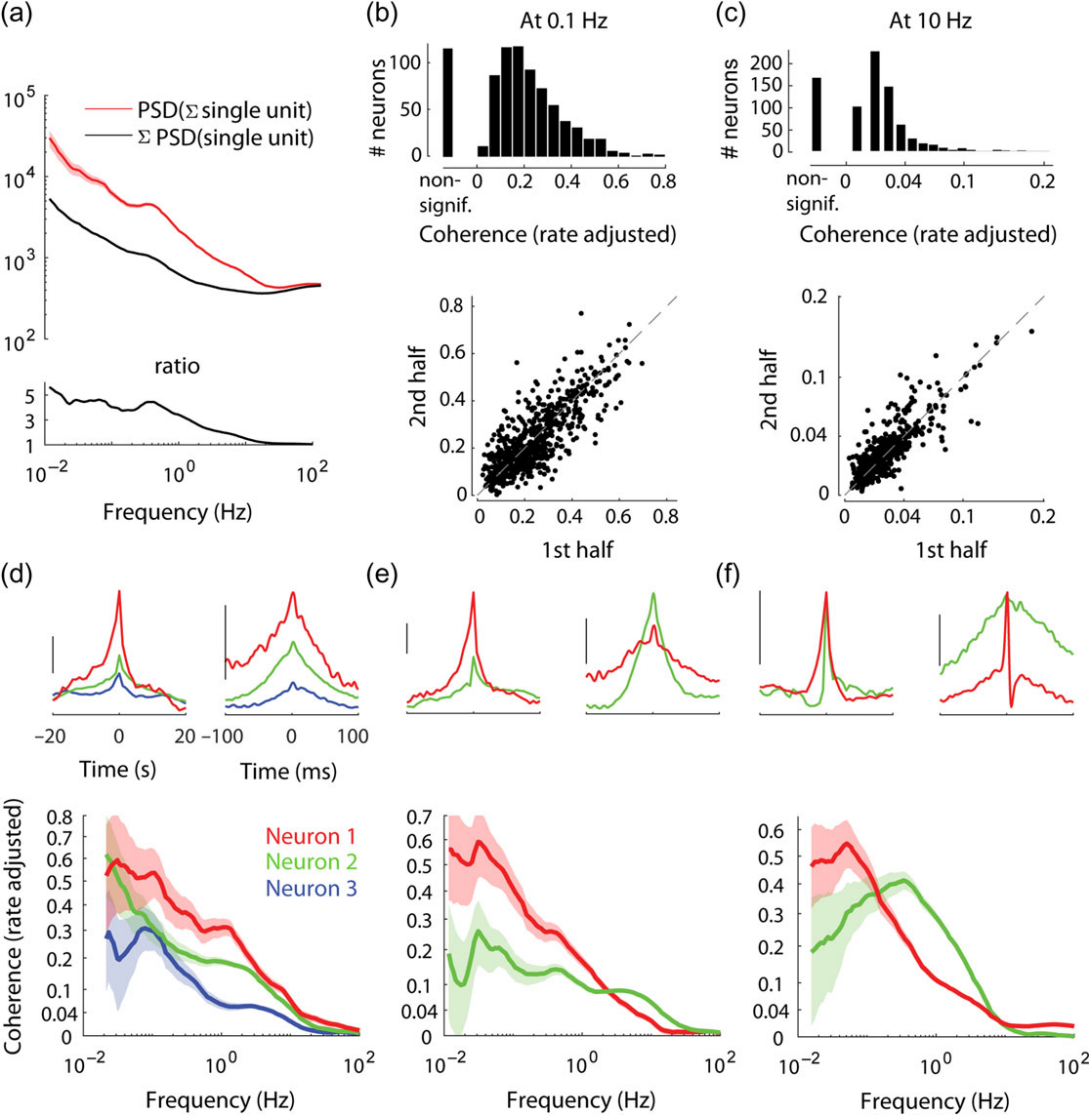
Frequency-resolved population coupling. (*a*) *Top*: PSD of population rate and sum of PSDs of individual spike trains comprising the population rate, in an example recording. Bottom: the ratio between the two, indicating that in frequencies < 1 Hz the former is several-fold higher. (*b*) *Top*: Distribution of rate adjusted coherence with population rate at an example slow timescale frequency (0.1 Hz) across all neurons. Some neurons have no significant coherence (“non-signif.”). *Bottom*: rate adjusted coherence with population rate at 0.1 Hz, evaluated separately in first and second halves of the recordings, *R*^2^ = 0.63 (*P* < 10^−100^). (*c*) Same format as *b*, for fast time scale example frequency (10 Hz), *R*^2^ = 0.75 (*P* < 10^−100^). (*d*) *Top*: Time domain correlation between spike trains of 3 example simultaneously recorded neurons and the population rate, on slow and fast time scales (scale bar: median amplitude of the correlation across all neurons in the recording). *Bottom*: Rate adjusted coherence of each example neuron with population rate. (*e*) Two example simultaneously recorded neurons, where one (red) has high coherence with the population in low frequencies and low coherence in high frequencies, when compared with the other neuron (green), which exhibits the opposite behavior. Layout as in *d*. (*f*) Two example simultaneously recorded neurons whose relative strength of population coupling switches twice over the frequency range, furthermore one of the neurons (green) has a nonmonotonic coherence with population. Note that the time domain correlation with population rate of both neurons is of equal magnitude on both fast and slow timescales. Layout as in *d*. In *c*–*f* coherence values are shown using power function scaling, to make low values visible. In *d*–*f* shaded areas indicate 95% confidence intervals.

Time domain correlation between spike trains of single neurons and the population rate (Fig. 3d-f) was computed as previously described in (Okun et al. 2015). Specifically, we computed the inner product between the vectors representing the population rate and single unit spike train at different lags (using Matlab’s xcorr), and normalized it by the number of spikes of the single neuron. For fast timescale correlation, the vectors were at 1 ms resolution, and the single neuron spike train was smoothed with Gaussian of 12 ms halfwidth. For slow timescale correlation, the vectors were at 1 s resolution. In both cases the baseline (average values 800–1000 ms away from zero lag for fast timescale correlation, and average values 12–20 s away from zero lag for slow timescale correlation) was subtracted.

### Coherence Analysis

For analyzing the relationship between spike trains of individual units and population rate, the latter was obtained by summing all the spikes detected on all the shanks/tetrodes barring the one on which the single unit was recorded. For Neuropixels recordings, where the entire probe consists of one shank (with 374 recording sites over ~3.5 mm) this approach was not applicable. Instead, for each unit we have performed our analyses with population rate based on all spikes on the probe (except for those of the unit itself) and with population rate based only on spikes from recording sites >60 μm away from the location of the single unit. All results were almost identical for both conditions. The population rate typically was >100 spikes/s.

Coherence between population rate or pupil area and individual units was estimated, together with its confidence interval, using coherencysegpb function of the Chronux toolbox (estimating coherency using coherencysegcpb, where population rate was considered a continuous signal rather than spike count, produced identical results). We used theoretical, asymptotic confidence intervals as computed by Chronux (we have also found that it provides more stringent, i.e., wider, intervals than jackknife). As in the case of PSD estimation, coherence in different frequencies was estimated by breaking the entire recording into segments of appropriate length for each frequency.

There is a non-intuitive correspondence between the coherence of some continuous signal with a spike train and its coherence with the continuous spike intensity signal underlying the spike train (e.g., the neuron’s membrane potential). Unlike the more familiar case of coherence between a pair of continuous processes, coherence between a continuous process and a point process (such as a spike train of a neuron) depends on the PSD and the rate of the latter. This (mathematically unavoidable) fact has two implications. First, the 1/f profile of firing rate PSD implies that coherence of the spike train with population rate falls with frequency, even when coherence between the underlying firing rate intensity and the population rate does not (Fig. S2c,d). Second, because coherence depends on the rate of the spike train, 2 neurons whose firing rate intensities are exactly proportional but unequal do not have the same coherence with population rate. To account for this second issue of rate dependence, we use a correction factor to produce a coherence which reflects a firing rate of 1 spike/s, rather than the actual firing rate of the neuron.

More formally, let y(t) be a continuous process, n(t) a spike train of a single neuron and λ(t) the underlying firing rate, that is, n(t) is a doubly stochastic Poisson process with intensity λ(t). It holds that

(1) $$C_{\mathrm{yn}}\left(\omega \right)=C_{y\lambda }\left(\omega \right){\left(1+\mu /S_{\lambda \lambda }\left(\omega \right)\right)}^{-1/2}$$

where, $C_{\mathrm{yn}}$ and $C_{y\lambda }$ denote the coherence between y(t) and n(t) or λ(t), μ is the mean rate of n(t), and $S_{\lambda \lambda }$ is the power spectrum of λ(t) (Aoi et al. 2015). From the above equation, it is clear that 2 spike trains with proportional but unequal rate intensities (i.e., if $\lambda_{1}\left(t\right)=\alpha \lambda_{2}\left(t\right)$ where α≠1) will have different values for coherence. This is not desirable, therefore instead of reporting the coherence between a spike train and the population rate, we report “rate adjusted coherence” which reflects the coherence that would have been measured if the neuron had a firing rate of 1 spike/s, that is, if its firing intensity was λ(t)/μ instead of λ(t), see Fig. S2a,b for an example. We use a correction factor of ${\left(1+\left(\mu -1\right)\mu /S_{\mathrm{nn}}\left(\omega \right)\right)}^{-1/2}$, as derived in Aoi et al. (2015), to obtain the rate adjusted coherence. The PSD of the spike train, used for the correction was estimated as described above.

The rate adjusted coherence still depends on the PSD of the spike train of the single unit. For instance, it is possible to have 2 intensity functions $\lambda_{1}\left(t\right)$ and $\lambda_{2}\left(t\right)$ with equal means of 1 spike/s, and equal coherence with y(t), but with different power spectra. In this case, Equation (1) implies that if $n_{1}$ and $n_{2}$ are spike trains with intensities $\lambda_{1}$ and $\lambda_{2}$, then $C_{yn_{1}}\left(\omega \right){\neq C}_{yn_{2}}\left(\omega \right)$ even though $C_{y\lambda_{1}}\left(\omega \right){=C}_{y\lambda_{2}}\left(\omega \right)$. Here, we did not attempt to remove this dependence, which would have required an accurate estimate of $S_{\lambda \lambda }\left(\omega \right)$. In practice, $S_{\lambda \lambda }$ cannot be directly inferred from $S_{\mathrm{nn}}$ because the assumption that n(t) is a doubly stochastic Poisson process with intensity λ(t) does not hold. For example, the existence of refractory period in n(t) reduces the power in all low frequencies in $S_{\mathrm{nn}}\left(\omega \right)$ (Bair et al. 1994; Rivlin-Etzion et al. 2006). Furthermore, the spiking of actual neurons is driven by changes in the subthreshold membrane potential $V_{m}\left(t\right)$ which is rather distinct from λ(t), as exemplified in Fig. S2. Of note, this discussion primarily applies to high frequencies, whereas in low frequencies μ is significantly lower than $S_{\lambda \lambda }\left(\omega \right)$ (or $S_{\mathrm{nn}}\left(\omega \right)$) and thus the discrepancy between $C_{\mathrm{yn}}\left(\omega \right)$ and $C_{y\lambda }\left(\omega \right)$ is minor (see Equation 1).

To compare the strength of population coupling of 2 simultaneously recorded neurons across all timescales, we have compared their rate adjusted coherences in the following specific frequencies: 0.01, 0.03, 0.1, 0.32, 1, 3.2, 10, 32, and 100 Hz. If the null hypothesis that first neuron has higher rate adjusted coherence in these frequencies could be rejected at *P* ≤ 0.001 (after using Bonferroni correction for performing 9 comparisons), and the reverse null hypothesis could also be rejected with *P* ≤ 0.001, the 2 were considered as (a positive) example of a simultaneously recorded pair of neurons where neither neuron dominated the other across all frequencies.

### Phase Analysis

**Figure 4.**
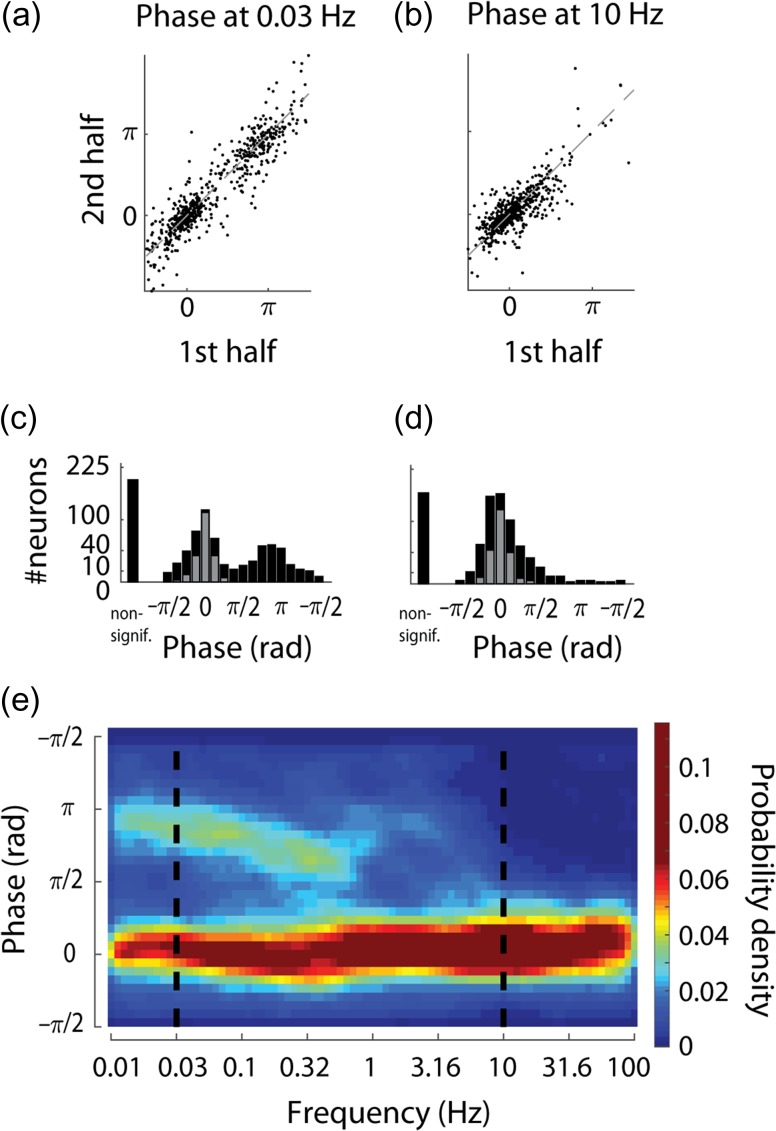
Phase of population-coupling. (*a*, *b*) Phase evaluated separately in first and second halves of the recordings, indicating that it is a conserved property for most neurons. Average absolute discrepancy between the 2 halves: 0.44 ± 0.43 rad and 0.34 ± 0.39 rad (mean and standard deviation for *n* = 582 and *n* = 610 neurons with statistically significant phase preference at 0.03 and 10 Hz, correspondingly). Explained circular variance: 0.79 and 0.54 (*P* < 10^−16^). (*c*, *d*) Distribution of the preferred phase of firing of individual neurons with respect to population rate at example frequencies (0.03 and 10 Hz). Some neurons have no significant coherence or phase preference (“non-signif.”); gray: neurons for which the phase was not significantly (i.e., *P* > 0.05) different from 0. (*e*) Pseudocolor histogram of phase preference with respect to population rate across 0.01–100 Hz. Dashed lines indicate the 2 example frequencies shown in *a*–*d*.

Phase of spiking of single units with respect to population rate or pupil area was estimated using the same Chronux toolbox functions used to estimate the coherence (see above). As in the case of PSD estimation, phase in different frequencies was estimated by breaking the entire recording into segments of appropriate length for each frequency. After the phase in each segment was estimated, circular mean and standard deviation were computed. If the distribution of phases (across the segments) had no statistically significant (at *P* ≤ 0.05) mean, no phase was assigned (e.g., the nonsignificant neurons in the histogram in Fig. 4c,d).

All phases are specified with respect to the population rate, for example, a phase of −π/4 means that the single unit lags behind the population rate, whereas a phase of π/6 means that the unit leads it.

Our MATLAB code for the estimation of PSD, coherence and phase, following the above described procedures, is publicly available on github.com/m-okun/FrequencyDomainPopulationAnalysis.

### Linearity and Logarithmicity Indices

For a continuous, nonconstant function f(x) defined on an interval [a,b] (0<a<b), consider the following expression:

$$log\frac{V_{b}^{k}f\left(x\right)}{V_{k}^{a}f\left(x\right)},$$

where, $\overset{c}{\underset{d}{⋁}}f\left(x\right)$ denotes the total variation of f(x) on the interval [c,d]. We define the linearity index of f(x) as the value of this expression for k=(a+b)/2. Similarly, the logarithmicity index is the expression’s value for $k=\sqrt{\mathrm{ab}}$. The rationale for these definitions is that for a function changing on a linear scale, total variation in the first and second halves of [a,b] is expected to be of comparable magnitude. Thus, linearity index is close to 0 for functions changing on linear scale (the function itself does not have to be linear, e.g., sin⁡(x) on any sufficiently long interval), positive for supralinear functions, and negative for sublinear functions. On the other hand, for a function changing on a logarithmic scale, the total variation in $\left[a,\sqrt{\mathrm{ab}}\right]$ and $\left[\sqrt{\mathrm{ab}},b\right]$ intervals is expected to be of comparable magnitude, thus its logarithmicity index would be close to 0 (while its linearity index would be negative).

**Figure 5.**
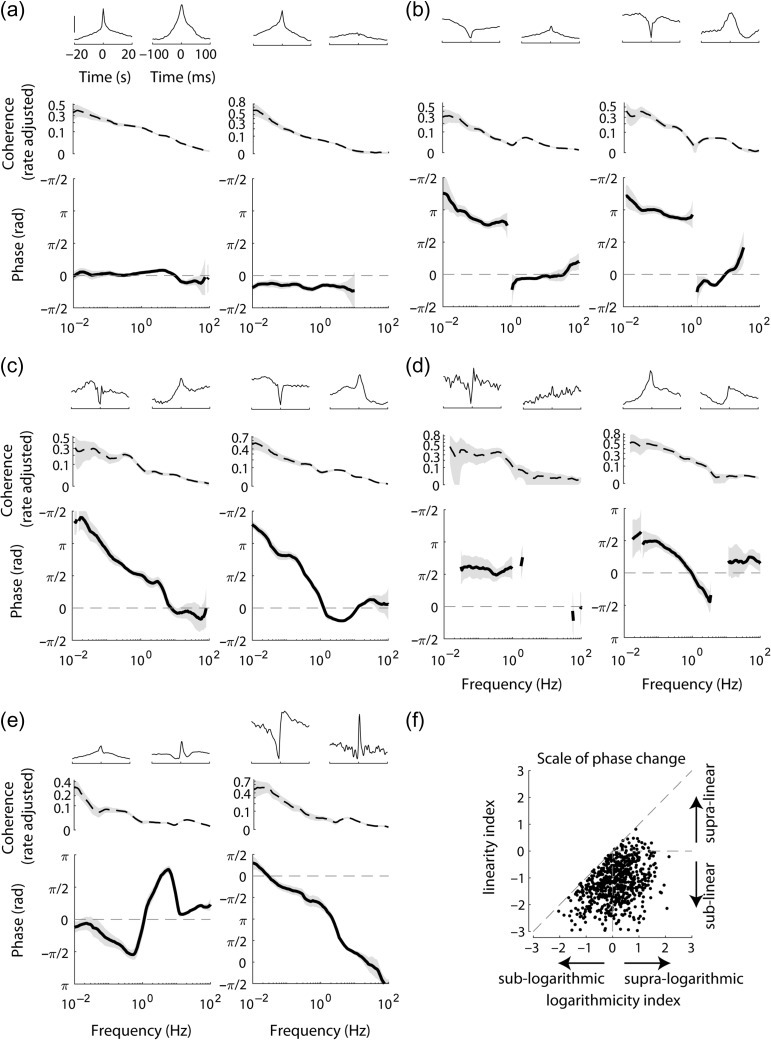
Population coupling phase spectrum. (*a*) Examples of neurons whose firing has phase preference close to 0 with respect to population rate. In the second example the phase is at the same time significantly distinct from 0. *Top*: time domain correlation between the neuron and population rate on fast and slow timescale (scale bar: median amplitude of the correlation across all neurons in the corresponding recording). *Middle*: rate adjusted coherence with population rate. *Bottom*: phase spectrum. (*b*) Examples of neurons with sharp transition between ~π phase in infraslow frequencies and ~0 phase preference in high frequencies, dividing the frequency range into 2 clear subdomains. (*c*) Examples of neurons whose phase preference is close to π in the infraslow frequencies and gradually becomes close to 0 in high-frequency range. (*d*, *e*) Additional examples of observed phase spectra behaviors. Panels *b*–*e* use the same format as *a*, shaded areas in *a*–*e* indicate 95% confidence intervals, *y*-axis of coherence plots uses power function scaling, to make low values visible. (*f*) Logarithmicity index versus linearity index (see Materials and Methods) of the longest continuous interval of the phase spectrum of each neuron.

For empirically measured f(x), total variation is contaminated by measurement noise. To avoid this problem, and relying on the fact that phase functions were either monotonic or had just a few extremum points (typical examples shown in Fig. 5), we used the following expression instead of the one given above:

(2) $$log\frac{\mathrm{diam}{\left\{f\left(x\right)\right\}}_{b\geq x\geq k}}{\mathrm{diam}{\left\{f\left(x\right)\right\}}_{k\geq x\geq a}}$$

$\mathrm{diam}{\left\{f\left(x\right)\right\}}_{d\geq x\geq c}$ denotes the diameter of the set ${\left\{f\left(x\right)\right\}}_{d\geq x\geq c}$ (except for cases when f(x) wraps around ±π, this is equal to maxf(x)−minf(x)). For each neuron, equation (2) was evaluated using the longest continuous interval of frequencies over which phase was well-defined (i.e., it had no frequencies in which coherency with population rate was not statistically significant). Neurons for which such interval spanned less than an order of magnitude were excluded from the analysis.

## Results

To examine the intrinsic spiking dynamics of single cortical neurons on timescales extending to tens of seconds and minutes we used multisite silicon probes to record the activity of neuronal populations in the frontal cortex. All the recordings were performed using chronically implanted probes (Okun et al. 2016; Jun et al. 2017). We used 16- and 32-channel Neuronexus probes in 6 animals and 374-channel Neuropixels probes in 2 additional animals. The recordings lasted 1.5–3 h in head-fixed mice, standing or sitting in a plastic tube (22/26 recordings, 730/775 neurons), and 4–8 h in freely behaving mice residing in their home cage (4/26 recordings, 45/775 neurons).

### Single Neurons Show Dynamics at Multiple Timescales

On fast timescales the spiking dynamics of cortical neurons can be summarized by the ISI distribution. The characteristic irregular firing of cortical neurons results in ISIs varying by several orders of magnitude (Softky and Koch 1993), with some neurons also exhibiting ISI histogram peaks indicating rhythmicity at particular frequencies (Fig. 1*a*).

A neuron’s ISI distribution was not on its own sufficient to account for the structure of its spike train at long timescales. Indeed, visual inspection shows that cortical firing rates typically fluctuate on timescales of minutes or more (Fig. 2*b*), longer than almost all ISIs of neurons with firing rates ≥1 spike/s. Synthetic spike trains created by randomly reshuffling the original ISIs did not have this slow timescale dynamics (Fig. 2*c*). The discrepancy between actual and ISI-shuffled data could be summarized by the spike count Fano factor: the variance divided by the mean of spike counts in bins of prescribed temporal duration (Fig. 2*d*). For bins of short duration (1–100 ms), Fano factors were close to 1, and the Fano factors of the original and shuffled data were similar. However, for bins of 1–100 s, the Fano factors of actual data were several-fold higher. Across all analyzed neurons (*n* = 775), the Fano factor of the number of spikes in 1024 ms bins was 1.6 times higher in the actual data compared with ISI-shuffled trains, and with 16 384 ms bins it was 4.8 times higher (for a summary across all bin sizes see the error of ISI model in Fig. 1*e*, below).

Although the ISI distribution could not alone capture the infraslow porion of a cell’s spiking dynamics, the combination of ISI distribution and spike train PSD provided a good approximation. Because our recordings lasted several hours, we were able to compute power spectra down to very low frequencies, where the PSD values were much higher than for fast frequencies, indicative of infraslow dynamics (Fig. 1*a*). We devised an algorithm that generates synthetic spike trains with prespecified PSD and ISI distributions (see Materials and Methods). The slow-timescale firing dynamics of these synthetic spike trains was visually similar to the original data (compare Fig. 1*b* with Fig. 2*b*) and closely matched the observed Fano factors over multiple timescales (Fig. 1*c*), as expected from the analytical relationship between Fano factor and autocorrelation of a stationary spike train (Teich et al. 1997). The full model was better than models that used either PSD or ISI distribution independently (Fig. 1*d*). At slow timescales (e.g., 1 min; Fig. 1*e*), Fano factors predicted from PSD alone are significantly closer to the actual values than ISI-based predictions, but still not as accurate as the full model. For fast timescales (e.g., 100 ms), ISIs predict spike count accurately, but the PSD alone is insufficient (Fig. 1*e*). The full model respects both constraints, and as a result provides predictions that are significantly better than either ISI distribution or PSD alone (e.g., for 1-min bins its average error is 47%, compared with 1072% and 305% for ISI only and PSD only models, Fig. 1*d*,*e*). These results also demonstrate the major contribution of slow dynamics to spiking variability in the cortex.

Cortical neurons are diverse in their intrinsic dynamics. This diversity is well characterized at short timescales by differences in spike regularity (Maimon and Assad 2009) and by the differing propensity of neurons to emit complex-spike bursts (McCormick et al. 1985; de Kock and Sakmann 2008), but dynamical diversity at slow timescales is largely unexplored. To address this question, we observed that the PSD of most neurons had power-law profile over the 0.01–1 Hz range, with the exponent significantly different between neurons (Fig. S3a). Fitting spike train power with a $\mathrm{const}/f^{\beta }$ function over 0.01–1 Hz revealed that the power-law exponent β covered a range of 0.39 ± 0.19 (mean and standard deviation for *n* = 775 neurons), and was conserved when fit separately for the first and second halves of each recording (Fig. 1*f*; *R*^2^ = 0.69 overall; median *R*^2^ of individual recordings = 0.63; *P* <0.05 in each recording). The power law exponent was unrelated to mean firing rate and weakly related to bursting (Fig. S3b–d). We conclude that cortical neurons are diverse in the strength of their infraslow firing rate fluctuations, and that the structure of these fluctuations can be summarized, to first approximation, by the PSD slope β.

### Population Coupling Strength is Unrelated at Fast and Slow Timescales

To understand how the slow dynamics of individual neurons was related to that of the entire population, we started by considering how individual neurons are related to the population rate—the summed rate of all spikes detected on the probe. At short timescales, neurons vary continuously in the strength of their coupling with population rate (Okun et al. 2015). To characterise the relation of neurons to the population across multiple timescales, we extended this analysis into the frequency domain. Analysis in frequency domain does not suffer from the inherent ambiguity of time domain analysis, where correlations computed using a time bin of a specific duration reflect co-modulation not just on the scale of the bin, but also on all slower timescales (Brody 1999).

The PSD of population rate had 1/f profile, similar to the profile of PSDs of single neuron spike trains. In high frequencies the value of the PSD of any spike train is dominated by the firing rate term (see the section on single spike train analysis in Materials and Methods), therefore in high frequencies PSD of population rate was close to the sum of PSDs of all the individual spike trains that together comprise the population rate. However, in all frequencies <1 Hz, the population rate PSD was several fold higher than the sum of PSDs of the individual spike trains (Fig. 3*a*, Fig. S4). As the PSDs of independent neurons would add linearly, this result indicates that infraslow fluctuations in firing rates of neurons were correlated in all these frequencies. To estimate the coherence between population rate and spike trains of specific neurons, we considered the former as a continuous function of time, and computed its “rate adjusted coherence” (Aoi et al. 2015) with the spike train of each neuron, which accounts for differences in mean firing rate between neurons (Fig. S2a,b; see Materials and Methods). To verify that this method provided a reliable measure, we estimated coherence separately in two halves of single recordings, obtaining similar estimates for coherence on both slow and fast timescales for most neurons (for 0.1 and 10 Hz, correspondingly: overall *R*^2^ = 0.63 and 0.75, median *R*^2^ of individual recordings = 0.59 and 0.61, *P* <0.05 in 19/26 and 22/26 recordings; Fig. 3*b*,*c*, see also Fig. S7).

Coherence analysis revealed widely diverse relationships to population rate, both between neurons, and between timescales within individual neurons. In all cases, coherence decayed to zero with increasing frequency. This behavior is a by-product of the point processes nature of spike trains and need not signify a decay in coherence between population rate and the membrane potential of individual neurons (Fig. S2c,d; see the section on coherence analysis in Materials and Methods). Importantly, the manner of this decay varied greatly between neurons (Fig. 3d-f). In some cases the relative strength of different neurons’ population coupling was conserved across frequencies (e.g., the red neuron in Fig. 3*d* has consistently larger coherence than the green or blue neurons). However, simultaneously recorded neurons often showed different rates of coherence decay: in 25% of simultaneously recorded pairs each neuron had a significantly stronger coherence in a subset of frequencies (Fig. 3*e*,*f*). Furthermore, some neurons’ coherence with population rate was nonmonotonic (15% of cells, e.g., green neuron in Fig. 3*f*). On average across neurons, rate adjusted coherence with population rate remained <0.5 in all our recordings, even in frequencies as low as 0.1–0.01 Hz (Fig. 3*b*, Fig. S5).

### Phase of Population Coupling Differs Across Timescales

Coherence is an indication of a constant phase relationship between 2 processes. Thus, if a neuron has high coherence with population rate at a given frequency, this means it fires at a reliable phase with respect to the population—but does not imply that this phase is zero. Phase analysis showed that most neurons had a stable phase preference with respect to population rate across halves of the recording on both slow and fast timescales (Fig. 4*a*,*b*, see also Fig. S7). It also revealed a major difference between phases of population coupling on slow and fast timescales, with out of phase activity several-fold more likely in the infraslow range (Fig. 4*a***–***e*). Specifically, at ≥10 Hz just 5% of cells had phase closer to π than to 0, whereas at ≤ 0.3 Hz this was the case for ≥ 28% of the cells (*P* < 10^−28^, *Z*-test for equality of 2 proportions). This however did not completely summarize a neuron’s phase preference: even within a mode, there remained significant correlation in a neuron’s precise phase from one half of the recording to the other (Fig. 4*a*), and at high frequencies phases also had reliable nonzero values across the 2 halves of the dataset (Fig. 4*b*,*d*). The preferred phase distribution in the infraslow frequencies was not symmetric: more neurons had phases between π/4 and 3π/4 (i.e., leading the population rate) than between −3π/4 and −π/4 (lagging the population rate, e.g., 16% vs. 10% at 0.1 Hz, *P* <0.001). The fact that neurons show an asymmetric phase distribution relative to their summed activity might seem contradictory, but was possible because neurons which lagged the population had higher firing rates compared with neurons which led it (4.5 ± 4.8 vs. 3.1 ± 4.8 spikes/s, for −3π/4 to −π/4 vs. π/4 to 3π/4, *P* <0.0001). In contrast to the phase preference of individual cells, the relative phase of population rate on different shanks or tetrodes (or on different segments of the Neuropixels probe) was close to 0 in all frequencies (Fig. S6), suggesting that variations in population coupling phase mainly differ within, rather than between local populations.

The phase spectra of individual neurons were diverse, and could show a complex dependence on frequency. According to the phase distribution histogram (Fig. 4*e*) one expects to find neurons’ phase preference to be close to either 0 or π at infraslow frequencies, and close to 0 at high frequencies. While many neurons conformed to this pattern (Fig. 5*a*, Fig. S7a), neurons that were anticorrelated with infraslow population rate differed in the dependence of phase preference on frequency: it was discontinuous, with clear subdomains and a drop in coherence in some neurons, but gradual in others (Fig. 5*b*,*c*, Fig. S7b,c). We also observed neurons whose phase preference did not fit the overall pattern, for example, having phase preference of ~π/2 in infraslow frequencies (Fig. 5*d*, Fig. S7d) or exhibiting altogether different behaviors (Fig. 5*e*, Fig. S7e).

Phase modulation seemed to occur on logarithmic rather than linear scale (Fig. 5*c***–***e*). To assess the rate of phase changes, we devised indices which quantify the linearity and logarithmicity of the phases (see Materials and Methods). The linearity index is 0 for phase changing on linear scale, and it is positive (negative) for phase changing on supra- (sub-) linear scale. Similarly, the logarithmicity index is 0 for phase changing on logarithmic scale, and it is positive (negative) for phase changing on supra- (sub-) logarithmic scale. We found that phase spectra were overwhelmingly changing sublinearly (just 4% had positive linearity index), whereas the logarithmicity index values were about equally distributed around 0 (logarithmicity index of 59% of the neurons was positive, Fig. 5*f*). We conclude that the phase between single neuron and population rates predominantly changes on a logarithmic scale with frequency.

The logarithmic rate of phase preference change implies that phase in nearby frequencies is similar. In other words, when only a small range of frequencies is considered (e.g., on linear scale), the phase is approximately constant, and thus to a first approximation single cell neuronal dynamics with respect to population rate is independent of timescale. To test this prediction we compared how well constant phase and linear phase models fit the phase preference in nearby frequencies (0.1 Hz vs. 0.03 Hz or 0.32 Hz). The former model corresponds to timescale-independent dynamics, the latter to a lead or lag by a fixed time interval between an individual neuron and the population rate. As predicted by the logarithmic rate of phase change, the constant phase model fit the data substantially better than the linear model (23% explained variance vs. no explained variance for 0.32 Hz, and 28% vs. 17% explained variance for 0.03 Hz, Fig. S8).

### Infraslow Dynamics Correlates With Pupil Diameter

**Figure 6.**
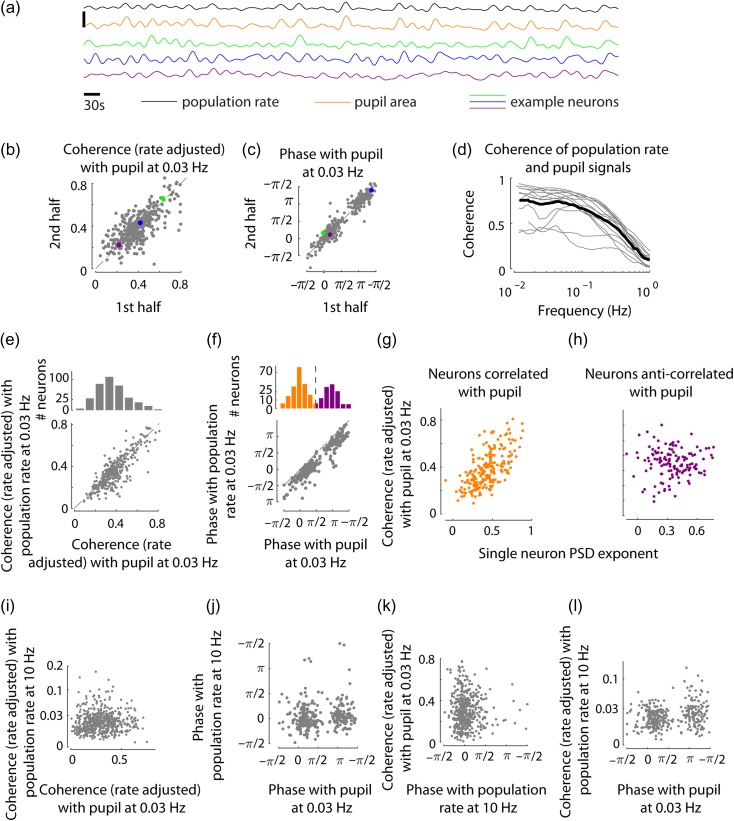
Global origin of infraslow dynamics. (*a*) Population rate, pupil area and spiking activity of example neurons during 1000 s portion of a recording. For presentation purposes only, signals were low-pass filtered below 0.05 Hz and *z*-scored, vertical scale bar is 5 standard deviations. (*b*, *c*) Magnitude (rate adjusted) and phase of coherency of individual neurons (*n* = 350, from 13 recordings) with pupil area signal, separately estimated from 2 halves of each recording, shown for an example frequency of 0.03 Hz. In *b*, *R*^2^ = 0.42 (*P* < 10^−50^). In *c*, average absolute discrepancy between the 2 halves: 0.34 ± 0.35 rad, explained circular variance: 0.88 (*P* < 10^−16^). Colored dots represent the example neurons shown in *a*. (*d*) Coherence between population rate and pupil area, in individual recordings (gray, *n* = 13 recordings in 5 animals) and in their average (black). (*e*, *f*) *Top*: Distribution of coherency magnitude (rate adjusted) and phase of individual neurons’ spiking with respect to the pupil area signal at 0.03 Hz. *Bottom*: magnitude and phase of coherency of individual neurons with pupil area vs their coherency with population rate. *R*^2^ = 0.67 (*P* < 10^−90^) in *e*, 0.85 explained circular variance (*P* < 10^−16^) in *f*. (*g*) The slope of power-law fit to the infraslow PSD portion (in 0.01–1 Hz) of each neuron was positively correlated with the rate adjusted coherence with pupil (at 0.03 Hz), for neurons whose phase with respect to pupil fluctuation was close to 0 (the left peak in the bimodal histogram in *f*, shown in orange; *r* = 0.64, *P* < 10^−9^). (*h*) No significant correlation was found between the PSD power-law slope and pupil coherence for neurons whose phase with respect to the pupil was close to π (the right peak in the bimodal histogram in *f*, shown in purple; *P* = 0.66). (*i*, *j*) Coherence (phase) of individual neurons with pupil signal on slow timescale (0.03 Hz) and their coherence (phase) with population rate on fast timescale (10 Hz) are uncorrelated (*P* = 0.85, Spearman correlation in *i*, *P* = 0.31, circular correlation in *j*). (*k*) Fast timescale phase was uncorrelated with coherence with the pupil (*P* = 0.77). (*l*) A significant correlation between phase with pupil at 0.03 Hz and coherence with population rate at 10 Hz (*P* < 0.001) was observed, where neurons anticorrelated with the pupil were more coherent with population rate on fast timescales.

Head-fixed mice, such as those we recorded here, show fluctuations in alertness levels over time. To address the degree to which the infraslow dynamics we observed could relate to alertness, we monitored the animals’ pupil area in a subset of head-fixed recordings (Fig. 6*a*). At 0.03 Hz, 65% (350/541) of the neurons were significantly coherent with the pupil area signal, and the magnitude of this coherence was consistent when estimated from separate halves of the recording (Fig. 6*b*; *P* <0.01 in 10/13 recordings, the median percentage of variance in one half of the data explained by the other half across recordings: 41%). Phase preferences were similarly stable, and the phase distribution had 2 clear peaks ~π rad apart (Fig. 6*c*), consistent with the existence of 2 populations positively and negatively coupled to arousal (Stringer et al. 2018).

Next, we considered how individual neurons’ coupling to the pupil and to the local population rate are related. Visual inspection of population rate and the pupil area signals suggested the two are similar in the infraslow range (Fig. 6*a*), which was confirmed by coherency analysis showing that the two signals were highly coherent in frequencies ≤0.1 Hz (Fig. 6*d*). Correspondingly, individual neurons’ coherence with pupil area closely matched their coherence with population rate (Fig. 6*e*). Phases with respect to the population rate and the pupil area were also closely matched; a consistent gap between the two (which at 0.03 Hz constituted 0.78 ± 0.51 rad) indicates that neuronal spiking preceded the pupil signal (Fig. 6*f*).

Coupling to pupil fluctuations was related to the infraslow dynamics of firing of individual neurons. We observed that the power-law exponent β obtained from fitting the spike train power with a $\mathrm{const}/f^{\beta }$ function (Fig. 1*f*) correlated with pupil coherence across the recorded neurons (*r* = 0.43, *P* <10^−9^). Interestingly, this correlation was highly significant only for neurons firing in phase with the pupil fluctuations, and insignificant for the antiphase neurons (Fig. 6*g*,*h*).

Population coupling on fast and slow timescales were largely unrelated: we observed no relationship between coherence with population rate on fast timescales and coherence with the pupil signal (*P* = 0.85, Fig. 6*i*; *P* >0.2 in each individual recording, Spearman correlation) and no relationship between the phases (*P* = 0.31, Fig. 6*j*, *P* >0.17 in each individual recording, circular correlation). Similarly, no significant relationship between fast timescale phase and coherence with the pupil was observed (*P* = 0.77, Fig. 6*k*), although phase with pupil did show a statistically significant relation to fast timescale coherence with the population (Fig. 6*l*, this might be due to some subclasses of cortical neurons differing in their fast and slow timescale population coupling properties). Considered together, these observations are consistent with the idea that population coupling on slow timescales is controlled by separate mechanisms from the local synaptic inputs driving fast timescale population coupling.

## Discussion

We used frequency domain analysis to examine the activity of neuronal populations in mPFC across frequencies spanning 4 orders of magnitude (0.01–100 Hz). Our findings point to a fundamental difference between fast and infraslow timescale cortical dynamics. The strengths of a neuron’s population coupling at fast and slow timescales were unrelated; furthermore, at fast timescales nearly all neurons fired at preferred phases close to 0 relative to population rate, whereas at slow timescales the phase distribution was bimodal, with preferred phases of ~30% of the neurons closer to π. Population coupling in infraslow, but not fast frequencies reflected coupling to brain-wide arousal signal (pupil area). While these general rules held for most neurons, a great diversity of fine-detailed behaviors was seen within local populations, for example regarding the slow-timescale dynamics as captured by a neuron’s power spectrum, and the way its coherency with population rate depended on frequency.

The difference between population coupling at fast and slow timescales likely indicates different mechanisms driving these types of coupling. Fast timescale dynamics reflects local synaptic activity (Haider and McCormick 2009), and the fast-timescale population coupling of individual neurons is correlated with the number of the local synaptic connections they receive (Okun et al. 2015). In contrast, infraslow dynamics correlates with global, brain-wide phenomena related to arousal, which are controlled at least in part by neuromodulatory inputs (McGinley et al. 2015; Reimer et al. 2016); a similar mechanism has been suggested for the global component of resting state fMRI measurements (Scholvinck et al. 2010; Wong et al. 2013; Turchi et al. 2018). The fact that a neuron’s population coupling on fast and slow timescales were uncorrelated therefore suggests that the degree to which a neuron’s firing is controlled by global brain states is unrelated to its local connectivity; for instance, a neuron weakly affected by neuromodulatory tone could still be strongly wired into the local network. The hypothesis that fast and slow population coupling arise through different mechanisms is supported by observations of neurons whose phase with population rate had discontinuous subdomains in high and low frequencies, as one would expect to see if the slow but not fast fluctuations are produced by a mechanism that suppresses the firing of these neurons while elevating the population rate (Fig. S9). This hypothesis is also supported by the fact that only slow-timescale population coupling phases were bimodal, an observation consistent with previous work in mouse primary visual cortex (V1). Specifically, in a previous study in V1 we observed neurons with weak fast-timescale population coupling but only very few with negative fast-timescale coupling (Okun et al. 2015). Furthermore, finding two populations of neurons in V1 that couple negatively as well as positively to arousal (on slow-timescales) was reported by Vinck et al. (2015) and Stringer et al. (2018). Whereas the recording conditions in these works were not identical to ours (mice were recorded on a wheel, and in (Vinck et al. 2015) arousal was increased by a sensory stimulus), the combined evidence from these studies seems to suggest that the dynamical properties we have described are not unique to the deep layers of mPFC. On the other hand, based on analysis of Utah array recordings in primates, it was suggested that the structure of neuronal activity in cortical networks is preserved across timescales (Kiani et al. 2015); it is unclear if this discrepancy is due to difference in species, cortical areas, or other factors.

Most of our present day knowledge on infraslow cortical dynamics comes from fMRI studies of resting state activity (Buckner et al. 2013; Raichle 2015; Foster et al. 2016). Because fMRI provides a blood-oxygen-level dependent (BOLD) signal rather than a direct measure of neural activity, it is limited to measurements on infraslow timescales. Multiple studies have shown that the BOLD signal correlates with population rate (Logothetis et al. 2001; Ma et al. 2016; Mateo et al. 2017), although disagreements on the BOLD signal’s interpretation remain (e.g., see Winder et al., 2017). Our study provides an account of how individual neurons’ activities combine to produce infraslow fluctuations in population rate, and hence in BOLD (to the extent the two are correlated). The low-frequency power of the population rate was 2–5 times larger than it would be if cells were independent of each other (Fig. 3*a*, Fig. S4). Because the recorded populations were spread over hundreds of micrometers, this increase would likely have been even higher if the recorded populations were concentrated in a smaller volume. We observed that the contribution of single neuron activity to the mesoscale signal is limited in two ways. First, for majority of neurons their coherence with population rate remained relatively low (typically between 0.2 and 0.4) even in the 0.01–0.1 Hz range of frequencies (Fig. S5), and for some neurons coherence in this range was found to be even lower than for higher frequencies (Fig. 3*f*). Second, the infraslow fluctuations in firing rate of many neurons were partially or completely out of phase with the population (Fig. 4). The present study is limited to measurements of spiking activity in one cortical area (including in cases where several population rate signals from different shanks or parts of the silicon probe were compared), the coherency relationship between neurons and population rates of distal cortical areas remains to be elucidated, for example, see Mitra et al. (2018). Another potential caveat with linking the present work to resting state fMRI studies is the degree to which the activity we observed is in the pure resting state regime (Logothetis et al. 2009; Winder et al. 2017). While it is possible to find short intervals during which a mouse does not move, this is not the case for intervals longer than a few seconds (e.g., typically mice move their eyes every 5–10 s). Thus our results should not be viewed as describing pure spontaneous activity (an ideal which is impossible to achieve in practice for infraslow timescales in awake subjects), but pertain to the actual cortical dynamics which is partially driven by intrinsic behaviors.

At the single neuron level, power spectral analysis was consistent with scale-free dynamics in the infraslow frequency range (Fig. 1*a*, Fig. S3). Such dynamics is typical of neuronal activity on various spatial scales, from fMRI measurements to ion channels, e.g., (Lowen et al. 1997, 1999; He 2011), and elsewhere in biology, for example, organization of heart beats (Bassingthwaighte et al. 1994). Similar spectra have been reported for retinal and thalamic cells recorded in anesthetized cats (Teich et al. 1997; Lowen et al. 2001), and more recently in resting humans (Nir et al. 2008), with a mean power-law exponent of 0.45, close to the 0.39 value observed here (Fig. 1*f*, Fig. S3d). For an intuitive interpretation of this value consider that for a signal with power spectrum proportional to $1/f^{0.4}$, 66% of the total power slower than any chosen frequency ω is concentrated in frequencies ≤ω/2. As a result of these slow changes in firing rate of individual neurons, their spike count variance in minute bins was on average ~10-fold higher than what fast-timescale spiking dynamics alone (i.e., the ISI model) would predict (Fig. 1*e*). The interpretation and causes of such scale-free dynamics are controversial. Here we find that in neurons positively (but not negatively) correlated with arousal the power-law exponent is highly correlated with the strength of pupil coupling (Fig. 6*g*,*h*), implicating brain-wide neuromodulatory mechanisms. Another suggested possibility is that on slow timescales it is inherent to recurrently connected networks (Chaudhuri et al. 2018). Yet another possibility is that scale-free behavior could be caused by single-cell intrinsic mechanisms such as firing rate adaptation (Marom 2010; Xu and Barak 2017), which can result in a frequency-independent lead of ~0.2 rad of the output spiking over sinusoidal input currents with periods <1 Hz (Lundstrom et al. 2008; Pozzorini et al. 2013). These effects can build up across more complex networks: for example, when rat whiskers were stimulated by white noise on top of which sinusoidal modulation with 0.3–0.03 Hz frequency was added, barrel cortex neurons preceded the sinusoidal stimulus envelope by ~0.8 rad on average, while thalamic neurons were leading by less than half as much (Lundstrom et al. 2010). An alternative, recently proposed possibility is that infraslow firing rate fluctuations are driven by slow changes in ion concentrations (Krishnan et al. 2018). While the contribution of each of these mechanisms remains to be elucidated, it is likely that their effect on cortical dynamics is particularly complex on intermediate timescales (~1 Hz) where they interact with fast-timescale local synaptic activity.

## Supplementary Material

Supplementary Data

## References

[bhz023C1] Aoi MC, Lepage KQ, Kramer MA, Eden UT 2015 Rate-adjusted spike-LFP coherence comparisons from spike-train statistics. J Neurosci Methods. 240:141–153. 25460189 10.1016/j.jneumeth.2014.11.012 PMC4336674

[bhz023C2] Bair W, Koch C, Newsome W, Britten K 1994 Power spectrum analysis of bursting cells in area MT in the behaving monkey. J Neurosci. 14:2870–2892. 8182445 10.1523/JNEUROSCI.14-05-02870.1994 PMC6577471

[bhz023C3] Bassingthwaighte JB, Liebovitch LS, West BJ 1994 Fractal physiology. New York: American Physiological Society / Oxford.

[bhz023C4] Brody C 1999 Correlations without synchrony. Neural Comput. 11:1537–1551. 10490937 10.1162/089976699300016133

[bhz023C5] Buckner RL, Krienen FM, Yeo BTT 2013 Opportunities and limitations of intrinsic functional connectivity MRI. Nat Neurosci. 16:832–837. 23799476 10.1038/nn.3423

[bhz023C6] Burgess CP, Lak A, Steinmetz NA, Zatka-Haas P, Bai Reddy C, Jacobs EAK, Linden JF, Paton JJ, Ranson A, Schröder S, et al 2017 High-yield methods for accurate two-alternative visual psychophysics in head-fixed mice. Cell Rep. 20:2513–2524. 28877482 10.1016/j.celrep.2017.08.047 PMC5603732

[bhz023C7] Chan AW, Mohajerani MH, LeDue JM, Wang YT, Murphy TH 2015 Mesoscale infraslow spontaneous membrane potential fluctuations recapitulate high-frequency activity cortical motifs. Nat Commun. 6:7738. 26190168 10.1038/ncomms8738 PMC5101061

[bhz023C8] Chaudhuri R, He BJ, Wang XJ 2018 Random recurrent networks near criticality capture the broadband power distribution of human ECoG dynamics. Cereb Cortex. 28:3610–3622. 29040412 10.1093/cercor/bhx233 PMC6132289

[bhz023C9] de Kock CPJ, Sakmann B 2008 High frequency action potential bursts (≥100 Hz) in L2/3 and L5B thick tufted neurons in anaesthetized and awake rat primary somatosensory cortex. J Physiol. 586:3353–3364. 18483066 10.1113/jphysiol.2008.155580 PMC2538819

[bhz023C10] Ecker AS, Berens P, Keliris GA, Bethge M, Logothetis NK, Tolias AS 2010 Decorrelated neuronal firing in cortical microcircuits. Science. 327:584–587. 20110506 10.1126/science.1179867

[bhz023C11] Foster BL, He BJ, Honey CJ, Jerbi K, Maier A, Saalmann YB 2016 Spontaneous neural dynamics and multi-scale network organization. Front Syst Neurosci.. 10:7. 26903823 10.3389/fnsys.2016.00007 PMC4746329

[bhz023C12] Haider B, McCormick DA 2009 Rapid neocortical dynamics: cellular and network mechanisms. Neuron. 62:171–189. 19409263 10.1016/j.neuron.2009.04.008 PMC3132648

[bhz023C13] Harris KD, Henze DA, Csicsvari J, Hirase H, Buzsáki G 2000 Accuracy of tetrode spike separation as determined by simultaneous intracellular and extracellular measurements. J Neurophysiol. 84:401–414. 10899214 10.1152/jn.2000.84.1.401

[bhz023C14] Harris KD, Thiele A 2011 Cortical state and attention. Nat Rev Neurosci. 12:509–523. 21829219 10.1038/nrn3084 PMC3324821

[bhz023C15] He BJ 2011 Scale-free properties of the functional magnetic resonance imaging signal during rest and task. J Neurosci. 31:13786–13795. 21957241 10.1523/JNEUROSCI.2111-11.2011 PMC3197021

[bhz023C16] Huk A, Bonnen K, He BJ 2018 Beyond trial-based paradigms: continuous behavior, ongoing neural activity, and natural stimuli. J Neurosci. 38:7551–7558. 30037835 10.1523/JNEUROSCI.1920-17.2018 PMC6113904

[bhz023C17] Jun JJ, Steinmetz NA, Siegle JH, Denman DJ, Bauza M, Barbarits B, Lee AK, Anastassiou CA, Andrei A, Aydın Ç, et al 2017 Fully integrated silicon probes for high-density recording of neural activity. Nature. 551:232–236. 29120427 10.1038/nature24636 PMC5955206

[bhz023C18] Kiani R, Cueva CJ, Reppas JB, Peixoto D, Ryu SI, Newsome WT 2015 Natural grouping of neural responses reveals spatially segregated clusters in prearcuate cortex. Neuron. 85:1359–1373. 25728571 10.1016/j.neuron.2015.02.014 PMC4366683

[bhz023C19] Kraft AW, Mitra A, Bauer AQ, Snyder AZ, Raichle ME, Culver JP, Lee J-M 2017 Visual experience sculpts whole-cortex spontaneous infraslow activity patterns through an Arc-dependent mechanism. Proc Natl Acad Sci. 114:E9952–E9961. 29087327 10.1073/pnas.1711789114 PMC5699067

[bhz023C20] Krishnan GP, González OC, Bazhenov M 2018 Origin of slow spontaneous resting-state neuronal fluctuations in brain networks. Proc Natl Acad Sci. 115:6858–6863. 29884650 10.1073/pnas.1715841115 PMC6042137

[bhz023C21] Krumin M, Shoham S 2009 Generation of spike trains with controlled auto- and cross-correlation functions. Neural Comput. 21:1642–1664. 19191596 10.1162/neco.2009.08-08-847

[bhz023C22] Lak A, Okun M, Moss M, Gurnani H, Wells MJ, Reddy CB, Harris KD, Carandini M 2018 Dopaminergic and frontal signals for decisions guided by sensory evidence and reward value. bioRxiv. 411413.

[bhz023C23] Lecci S, Fernandez LMJ, Weber FD, Cardis R, Chatton J-Y, Born J, Luthi A 2017 Coordinated infraslow neural and cardiac oscillations mark fragility and offline periods in mammalian sleep. Sci Adv. 3:e1602026. 28246641 10.1126/sciadv.1602026 PMC5298853

[bhz023C24] Lepage KQ, Kramer MA, Eden UT 2011 The dependence of spike field coherence on expected intensity. Neural Comput. 23:2209–2241. 21671792 10.1162/NECO_a_00169

[bhz023C25] Logothetis NK, Murayama Y, Augath M, Steffen T, Werner J, Oeltermann A 2009 How not to study spontaneous activity. Neuroimage. 45:1080–1089. 19344685 10.1016/j.neuroimage.2009.01.010

[bhz023C26] Logothetis NK, Pauls J, Augath M, Trinath T, Oeltermann A 2001 Neurophysiological investigation of the basis of the fMRI signal. Nature. 412:150–157. 11449264 10.1038/35084005

[bhz023C27] Lowen SB, Cash SS, Poo M, Teich MC 1997 Quantal neurotransmitter secretion rate exhibits fractal behavior. J Neurosci. 17:5666–5677. 9221766 10.1523/JNEUROSCI.17-15-05666.1997 PMC6573209

[bhz023C28] Lowen SB, Liebovitch LS, White JA 1999 Fractal ion-channel behavior generates fractal firing patterns in neuronal models. Phys Rev E Stat Phys Plasmas Fluids Relat Interdiscip Topics. 59:5970. 11969579 10.1103/physreve.59.5970

[bhz023C29] Lowen SB, Ozaki T, Kaplan E, Saleh BEA, Teich MC 2001 Fractal features of dark, maintained, and driven neural discharges in the cat visual system. Methods. 24:377–394. 11466002 10.1006/meth.2001.1207

[bhz023C30] Lundstrom BN, Fairhall AL, Maravall M 2010 Multiple timescale encoding of slowly varying whisker stimulus envelope in cortical and thalamic neurons in vivo. J Neurosci. 30:5071–5077. 20371827 10.1523/JNEUROSCI.2193-09.2010 PMC6632796

[bhz023C31] Lundstrom BN, Higgs MH, Spain WJ, Fairhall AL 2008 Fractional differentiation by neocortical pyramidal neurons. Nat Neurosci. 11:1335–1342. 18931665 10.1038/nn.2212 PMC2596753

[bhz023C32] Ma Y, Shaik MA, Kozberg MG, Kim SH, Portes JP, Timerman D, Hillman EMC 2016 Resting-state hemodynamics are spatiotemporally coupled to synchronized and symmetric neural activity in excitatory neurons. Proc Natl Acad Sci USA. 113:E8463–E8471. 27974609 10.1073/pnas.1525369113 PMC5206542

[bhz023C33] Macke JH, Berens P, Ecker AS, Tolias AS, Bethge M 2009 Generating spike trains with specified correlation coefficients. Neural Comput. 21:397–423. 19196233 10.1162/neco.2008.02-08-713

[bhz023C34] Maimon G, Assad JA 2009 Beyond Poisson: increased spike-time regularity across primate parietal cortex. Neuron. 62:426–440. 19447097 10.1016/j.neuron.2009.03.021 PMC2743683

[bhz023C35] Mainen ZF, Sejnowski TJ 1995 Reliability of spike timing in neocortical neurons. Science. 268:1503–1506. 7770778 10.1126/science.7770778

[bhz023C36] Marom S 2010 Neural timescales or lack thereof. Prog Neurobiol. 90:16–28. 19836433 10.1016/j.pneurobio.2009.10.003

[bhz023C37] Mateo C, Knutsen PM, Tsai PS, Shih AY, Kleinfeld D 2017 Entrainment of arteriole vasomotor fluctuations by neural activity is a basis of blood-oxygenation-level-dependent “resting-state” connectivity. Neuron. 96:936–948.e3. 29107517 10.1016/j.neuron.2017.10.012 PMC5851777

[bhz023C38] McCormick DA, Connors BW, Lighthall JW, Prince DA 1985 Comparative electrophysiology of pyramidal and sparsely spiny stellate neurons of the neocortex. J Neurophysiol. 54:782–806. 2999347 10.1152/jn.1985.54.4.782

[bhz023C39] McGinley MJ, Vinck M, Reimer J, Batista-Brito R, Zagha E, Cadwell CR, Tolias AS, Cardin JA, McCormick DA 2015 Waking state: rapid variations modulate neural and behavioral responses. Neuron. 87:1143–1161. 26402600 10.1016/j.neuron.2015.09.012 PMC4718218

[bhz023C40] Meisel C, Klaus A, Vyazovskiy VV, Plenz D 2017 The interplay between long- and short-range temporal correlations shapes cortex dynamics across vigilance states. J Neurosci. 37:10114–10124. 28947577 10.1523/JNEUROSCI.0448-17.2017 PMC5647769

[bhz023C41] Mitra P, Bokil H 2007 Observed BrainDynamics. 1st ed Oxford; New York: Oxford University Press. ed.

[bhz023C42] Mitra A, Kraft A, Wright P, Acland B, Snyder AZ, Rosenthal Z, Czerniewski L, Bauer A, Snyder L, Culver J, et al 2018 Spontaneous infra-slow brain activity has unique spatiotemporal dynamics and laminar structure. Neuron. 98:297–−305.e6. 29606579 10.1016/j.neuron.2018.03.015 PMC5910292

[bhz023C43] Nir Y, Mukamel R, Dinstein I, Privman E, Harel M, Fisch L, Gelbard-Sagiv H, Kipervasser S, Andelman F, Neufeld MY, et al 2008 Interhemispheric correlations of slow spontaneous neuronal fluctuations revealed in human sensory cortex. Nat Neurosci. 11:1100–1108. 19160509 10.1038/nn.2177 PMC2642673

[bhz023C44] Okun M, Lak A, Carandini M, Harris KD 2016 Long term recordings with immobile silicon probes in the mouse cortex. PLoS One. 11:e0151180. 26959638 10.1371/journal.pone.0151180 PMC4784879

[bhz023C45] Okun M, Steinmetz NA, Cossell L, Iacaruso MF, Ko H, Barthó P, Moore T, Hofer SB, Mrsic-Flogel TD, Carandini M, et al 2015 Diverse coupling of neurons to populations in sensory cortex. Nature. 521:511–515. 25849776 10.1038/nature14273 PMC4449271

[bhz023C46] Pachitariu M, Steinmetz NA, Kadir SN, Carandini M, Harris KD 2016. Fast and accurate spike sorting of high-channel count probes with KiloSort. In: Advances in Neural Information Processing Systems. p. 4448–4456.

[bhz023C47] Palva JM, Palva S 2012 Infra-slow fluctuations in electrophysiological recordings, blood-oxygenation-level-dependent signals, and psychophysical time series. Neuroimage. 62:2201–2211. 22401756 10.1016/j.neuroimage.2012.02.060

[bhz023C48] Popa D, Popescu AT, Pare D 2009 Contrasting activity profile of two distributed cortical networks as a function of attentional demands. J Neurosci. 29:1191–1201. 19176827 10.1523/JNEUROSCI.4867-08.2009 PMC2667329

[bhz023C49] Pozzorini C, Naud R, Mensi S, Gerstner W 2013 Temporal whitening by power-law adaptation in neocortical neurons. Nat Neurosci. 16:942–948. 23749146 10.1038/nn.3431

[bhz023C50] Raichle ME 2015 The restless brain: how intrinsic activity organizes brain function. Philos Trans R Soc Lond B Biol Sci. 370:20140172. 25823869 10.1098/rstb.2014.0172 PMC4387513

[bhz023C51] Reimer J, McGinley MJ, Liu Y, Rodenkirch C, Wang Q, McCormick DA, Tolias AS 2016 Pupil fluctuations track rapid changes in adrenergic and cholinergic activity in cortex. Nat Commun. 7:13289. 27824036 10.1038/ncomms13289 PMC5105162

[bhz023C52] Rivlin-Etzion M, Ritov Y, Heimer G, Bergman H, Bar-Gad I 2006 Local shuffling of spike trains boosts the accuracy of spike train spectral analysis. J Neurophysiol. 95:3245–3256. 16407432 10.1152/jn.00055.2005

[bhz023C53] Rossant C, Kadir SN, Goodman DFM, Schulman J, Hunter MLD, Saleem AB, Grosmark A, Belluscio M, Denfield GH, Ecker AS, et al 2016 Spike sorting for large, dense electrode arrays. Nat Neurosci. 19:634–641. 26974951 10.1038/nn.4268 PMC4817237

[bhz023C54] Schmitzer-Torbert N, Jackson J, Henze D, Harris K, Redish AD 2005 Quantitative measures of cluster quality for use in extracellular recordings. Neuroscience. 131:1–11. 15680687 10.1016/j.neuroscience.2004.09.066

[bhz023C55] Scholvinck ML, Maier A, Ye FQ, Duyn JH, Leopold DA 2010 Neural basis of global resting-state fMRI activity. Proc Natl Acad Sci. 107:10238–10243. 20439733 10.1073/pnas.0913110107 PMC2890438

[bhz023C56] Schreiber T, Schmitz A 1996 Improved surrogate data for nonlinearity tests. Phys Rev Lett. 77:635–638. 10062864 10.1103/PhysRevLett.77.635

[bhz023C57] Siegle JH, López AC, Patel YA, Abramov K, Ohayon S, Voigts J 2017 Open Ephys: an open-source, plugin-based platform for multichannel electrophysiology. J Neural Eng. 14:045003. 28169219 10.1088/1741-2552/aa5eea

[bhz023C58] Softky WR, Koch C 1993 The highly irregular firing of cortical cells is inconsistent with temporal integration of random EPSPs. J Neurosci. 13:334–350. 8423479 10.1523/JNEUROSCI.13-01-00334.1993 PMC6576320

[bhz023C59] Stringer C, Pachitariu M, Steinmetz N, Reddy CB, Carandini M, Harris KD 2018 Spontaneous behaviors drive multidimensional, brain-wide population activity. bioRxiv. 306019. 10.1126/science.aav7893 PMC6525101 31000656

[bhz023C60] Teich MC, Heneghan C, Lowen SB, Ozaki T, Kaplan E 1997 Fractal character of the neural spike train in the visual system of the cat. J Opt Soc Am A Opt Image Sci Vis. 14:529–546. 9058948 10.1364/josaa.14.000529

[bhz023C61] Turchi J, Chang C, Ye FQ, Russ BE, Yu DK, Cortes CR, Monosov IE, Duyn JH, Leopold DA 2018 The basal forebrain regulates global resting-state fMRI fluctuations. Neuron. 97:940–952.e4. 29398365 10.1016/j.neuron.2018.01.032 PMC5823771

[bhz023C62] Vinck M, Batista-Brito R, Knoblich U, Cardin JA 2015 Arousal and locomotion make distinct contributions to cortical activity patterns and visual encoding. Neuron. 86:740–754. 25892300 10.1016/j.neuron.2015.03.028 PMC4425590

[bhz023C63] Weber F, Dan Y 2016 Circuit-based interrogation of sleep control. Nature. 538:51–59. 27708309 10.1038/nature19773

[bhz023C64] White BR, Bauer AQ, Snyder AZ, Schlaggar BL, Lee J-M, Culver JP 2011 Imaging of functional connectivity in the mouse brain. PLoS One. 6:e16322. 21283729 10.1371/journal.pone.0016322 PMC3024435

[bhz023C65] Winder AT, Echagarruga C, Zhang Q, Drew PJ 2017 Weak correlations between hemodynamic signals and ongoing neural activity during the resting state. Nat Neurosci. 20:1761–1769. 29184204 10.1038/s41593-017-0007-y PMC5816345

[bhz023C66] Wong CW, Olafsson V, Tal O, Liu TT 2013 The amplitude of the resting-state fMRI global signal is related to EEG vigilance measures. Neuroimage. 83:983–990. 23899724 10.1016/j.neuroimage.2013.07.057 PMC3815994

[bhz023C67] Xu T, Barak O 2017 Dynamical timescale explains marginal stability in excitability dynamics. J Neurosci. 37:4508–4524. 28348138 10.1523/JNEUROSCI.2340-16.2017 PMC6596665

