# Supplementary material for: Distinct Structure of Cortical Population Activity on Fast and Infraslow Timescales
Source: Cereb Cortex. 2019 Feb 23;29(5):2196–210. doi: 10.1093/cercor/bhz023 (PMC6458908; doi:10.1093/cercor/bhz023)
Supplement: Supplementary Data [file bhz023_supp_resubm2.pdf]

Supplementary figures

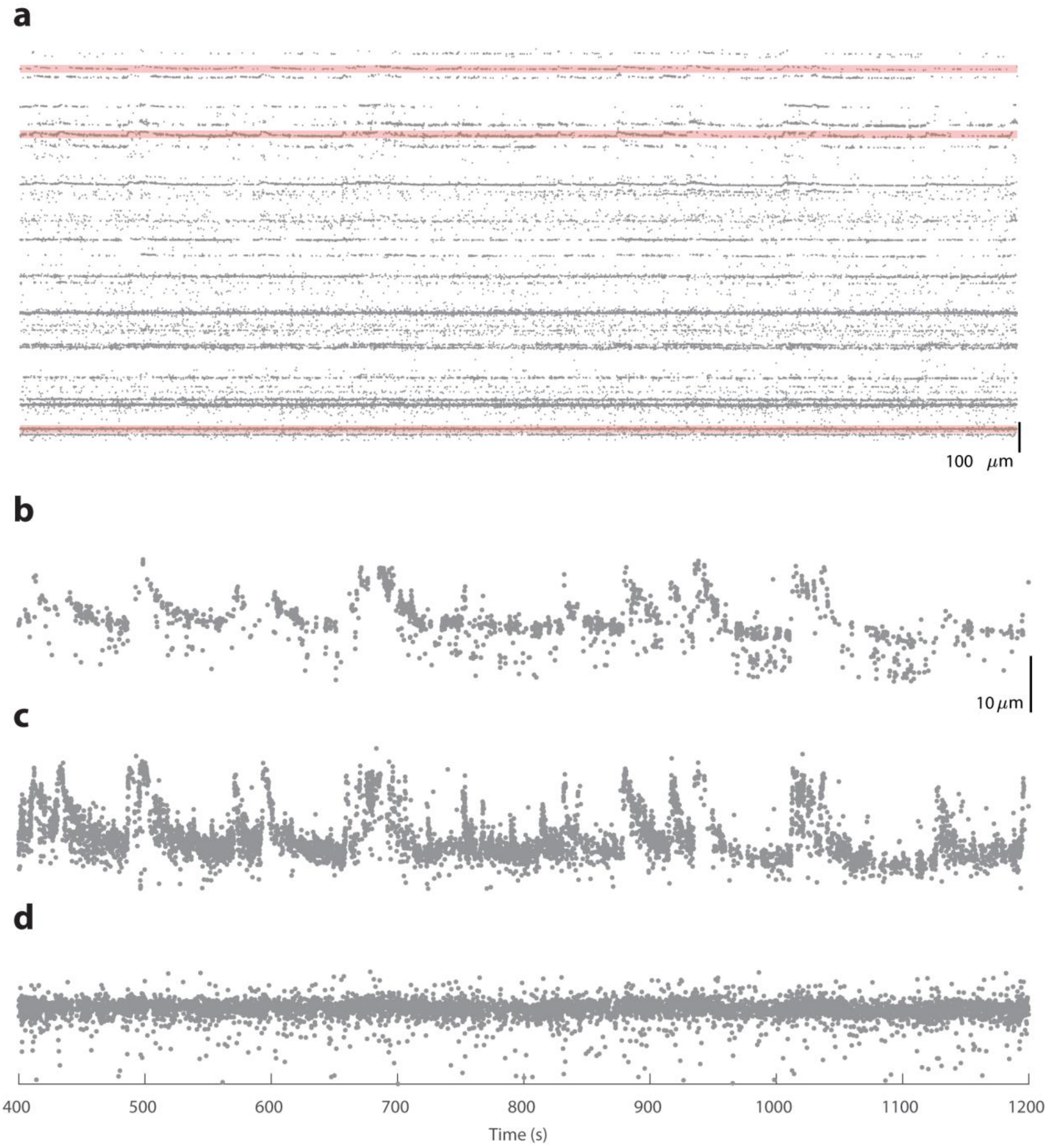

Figure S1. Drift detection in Neuropixels probe recordings. (a) Vertical position of each high-amplitude spike detected during 800 s portion of an example recording in the top 1.4 mm of a Neurpixels probe. **(b-d)** Three locations on the probe (highlighted in **a**), shown with a higher spatial resolution. In **b, c** drifts of 10-15 µm are clearly visible. The drift events are tens of seconds in duration and occur simultaneously at both locations. Drifts are not present at the third location shown in **d**, ~1 mm further down the probe.

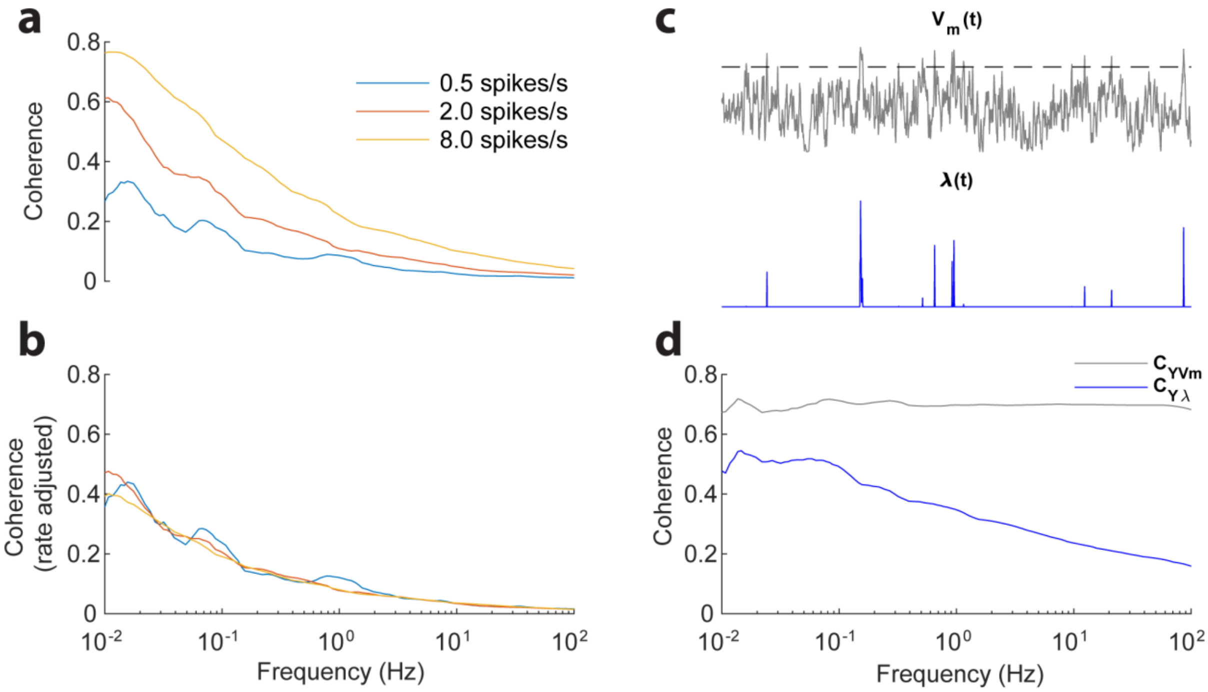

Figure S2. Estimating coherence with spike trains. (a) Spike trains with rates of 0.5, 2 and 8 spikes/s, and intensity of $a\lambda(t)$, where a controlled firing rate and $\lambda(t) \geq 0$ was an artificial signal with $1/f$ power, were generated. The coherence of the three spike trains with $\lambda(t)$ depends on their firing rate, although the coherence of their underlying intensity with $\lambda(t)$ is 1 in all three cases. **(b)** The rate adjusted coherence of the spike trains in **a** is similar. **(c,d)** Rate adjusted coherence, demonstrated in **b**, relies on the mathematical formalism of a doubly stochastic Poisson process, which does not apply to actual neurons where spikes are driven by membrane potential (V_m) fluctuations, and the spike generation mechanism is to a large extent reliable (Mainen and Sejnowski 1995). Yet, even for actual neurons one could think of a continuous firing rate $\lambda(t)$ that gives rise to the observed spike train. To a first approximation such $\lambda(t)$ is $V_m(t)$ transformed through a static non-linearity (determined by the spiking mechanism), as demonstrated by a synthetic example in **c**. Such transformation implies that coherence between any other signal $Y(t)$ and $\lambda(t)$ (denoted by $C_{Y\lambda}(\omega)$) deviates markedly from $C_{YVm}(\omega)$ (the coherence between $Y(t)$ and $V_m(t)$). In fact, one can show that in this case $C_{Y\lambda}(\omega) = const \cdot \sqrt{S_{VmVm}(\omega)/S_{\lambda\lambda}(\omega)} \cdot C_{YVm}(\omega)$, where $S_{VmVm}(\omega)$ and $S_{\lambda\lambda}(\omega)$ denote the PSD of $V_m(t)$ and $\lambda(t)$. In the example in **c,d**, a pair of artificial signals $Y(t)$ and $V_m(t)$ have a constant coherence of 0.7 across all frequencies, whereas the coherence between $Y(t)$ and $\lambda(t)$, derived from $V_m(t)$ via static nonlinearity, is no longer constant but falls with frequency, which is explained by the square root of the ratio between PSDs of $V_m(t)$ and $\lambda(t)$.

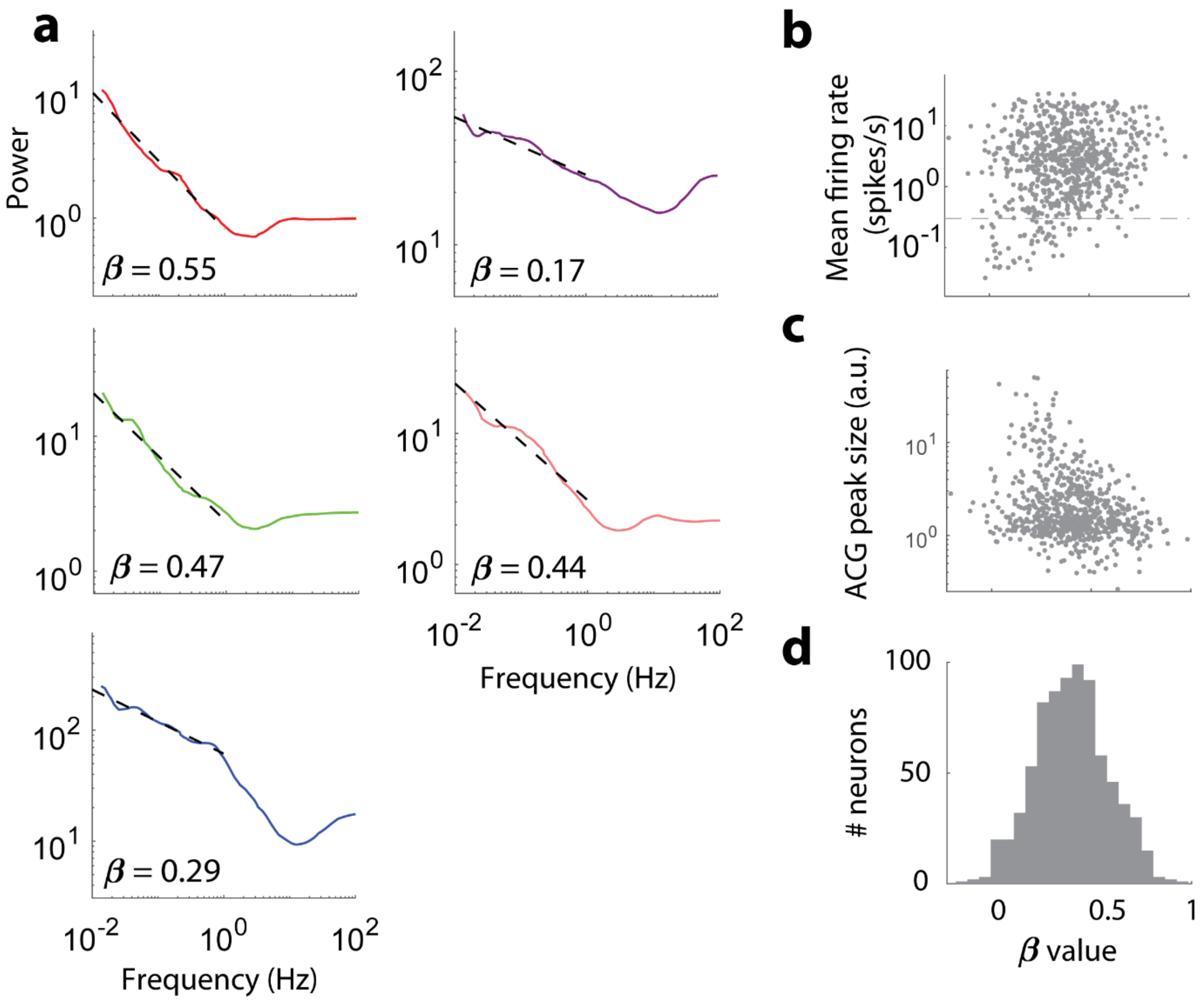

Figure S3. Power-law behaviour of infraslow power spectrum of spiking in cortical neurons. (a) The spike train power spectrum in the 0.01 – 1 Hz range was fitted with $const/f^{\beta}$ function. The fit for the five example neurons from Figure 1 is shown by a dashed line. **(b)** Power-law exponent shows no relationship to mean firing rate of the neurons, except for neurons with very low firing rate (where β is low owing to estimation bias, equally present in simulated data). For neurons with mean firing rate ≥ 0.3 spikes/s its correlation with β was low and insignificant: 0.03, P = 0.39 (Spearman correlation). **(c)** Power-law exponent is weakly correlated with burstiness (the ratio between the peak and baseline of a neuron's autocorrelogram), r = -0.23, P < 10^{-9} (Spearman correlation). **(d)** Distribution of the power-law exponent value across all the analysed neurons.

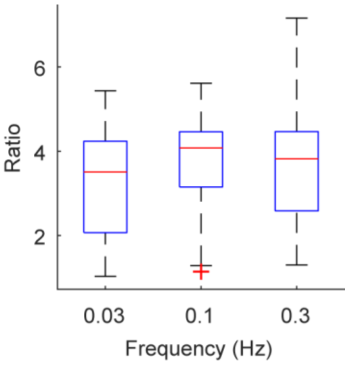

Figure S4. Increase in population rate PSD produced by neuronal coherence. The ratio between PSD of population rate and sum of PSDs of the firing rates of individual neurons that constitute it, averaged across all recordings (n = 26), at 0.03, 0.1 and 0.3 Hz. In these frequencies PSD of population rate was on average 3-4 times higher than what it would have been if the firing rates of the neurons were uncorrelated.

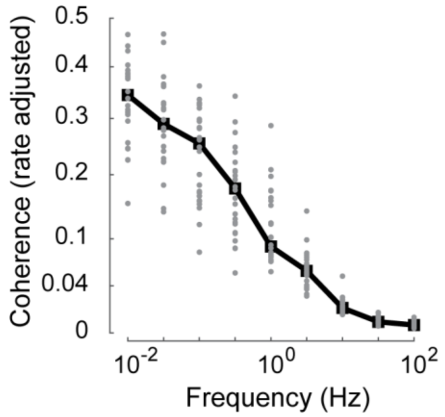

Figure S5. Average frequency-resolved population coupling. The value of rate adjusted coherence with population rate averaged across all neurons in each recording (grey points), and its median across all recordings (black).

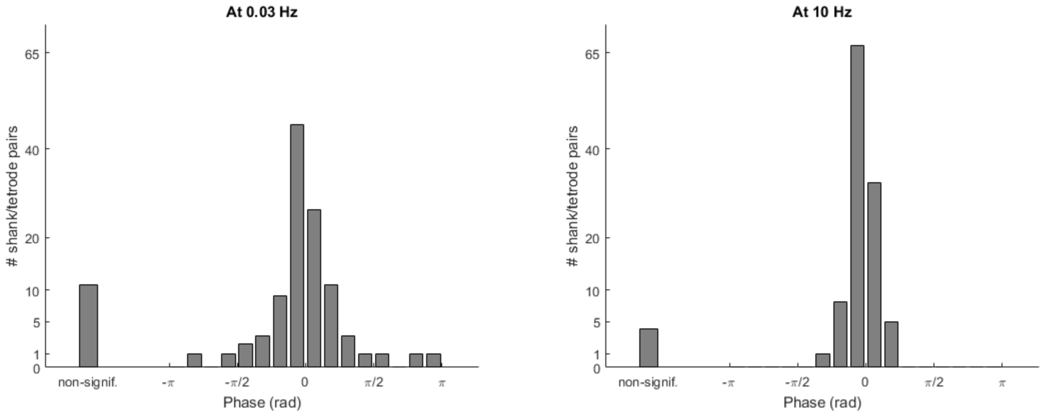

Figure S6. Phase between multiunit activity (MUA) on different shanks or tetrodes in the same recording. Phase between MUA signals is shown for 0.03 and 10 Hz for all pairs of shanks/tetrodes. Unlike phase distribution between population rate and individual neurons, the distribution of phases between MUA signals is unimodal in both infraslow and high frequencies (cf. Figure 4c-d).

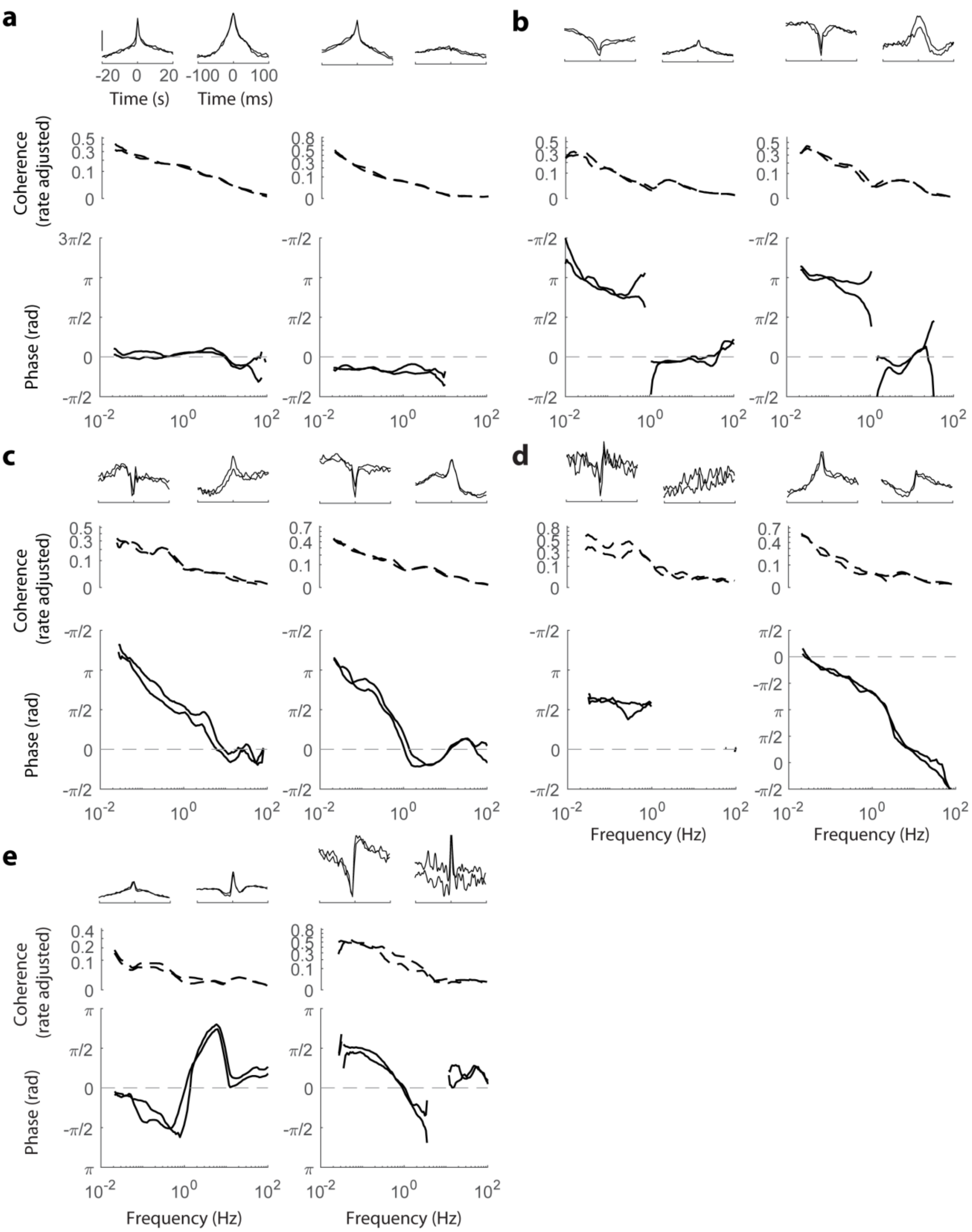

Figure S7. Population coupling phase spectrum. For each example neuron in Figure 5, time domain correlation between the neuron and population rate on fast and slow timescale (top), and its coherence (middle) and phase (bottom) with respect to population rate were evaluated in each half of the recording separately. The values in the two halves closely overlap.

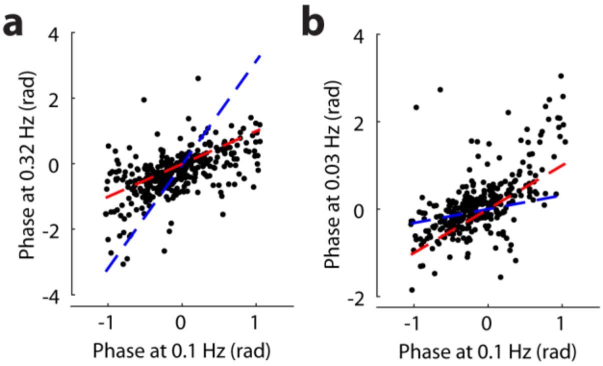

Figure S8. Comparison of linear phase and constant phase models. (a) Phase at 0.1 Hz vs phase at 0.32 Hz. Red dashed line indicates identity, corresponding to constant phase model, with $R^2 = 0.23$. Linear phase model is shown by blue dashed line, with $R^2 < 0$. **(b)** Phase at 0.1 Hz vs phase at 0.03 Hz. The dashed lines show the two models as in **a**. For constant phase model $R^2 = 0.28$, for linear phase model $R^2 = 0.17$. In **a, b** the comparison was limited to neurons whose phase at 0.1 Hz was sufficiently close to 0 (specifically, within 1 rad; using other intervals produced similar results).

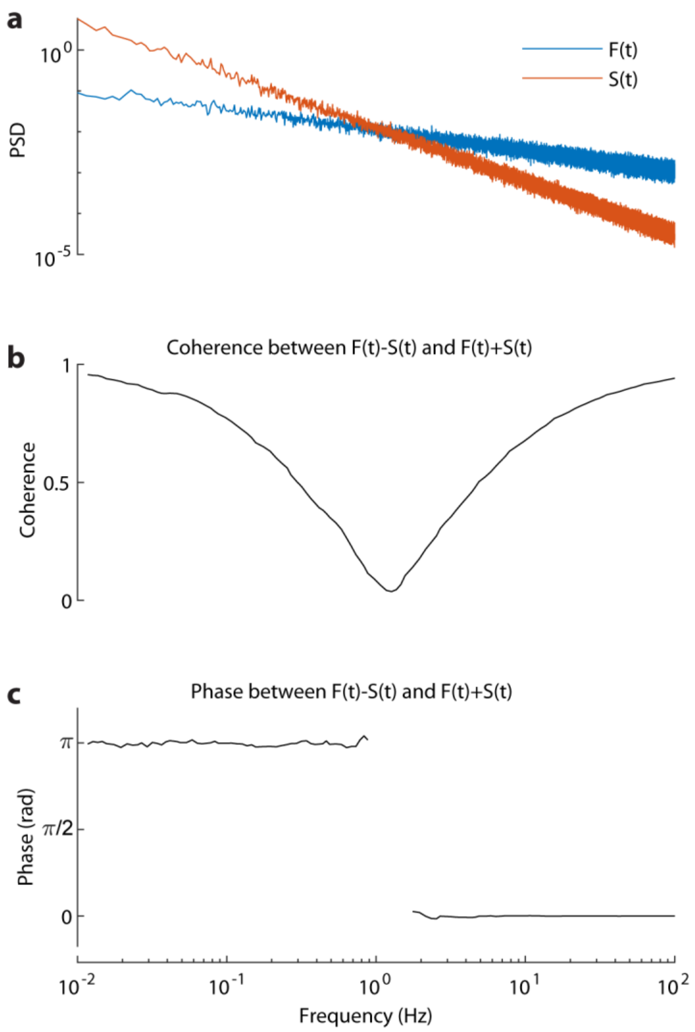

Figure S9. Example of discontinuous phase between two signals. (a) $F(t)$ is a ('fast') signal which has high power in high frequencies and low power in low frequencies compared to another ('slow') signal $S(t)$. For example, $F(t)$ could represent fast synaptic inputs generated by local processing in a cortical circuit, while $S(t)$ represents the slow brain-wide arousal signal. Here the two signals are taken to be independent. **(b)** Coherence between $F(t) - S(t)$ and $F(t) + S(t)$. The first signal represents activity of a neuron whose firing rate is suppressed by increased arousal, whereas the second signal represents the population rate, which increases with arousal. **(c)** Phase between $F(t) - S(t)$ and $F(t) + S(t)$. The values in **b, c** were estimated from 2 hours of simulated $F(t)$ and $S(t)$ signals.
